# Eosinophilic granulomatosis with polyangiitis – Advances in pathogenesis, diagnosis, and treatment

**DOI:** 10.3389/fmed.2023.1145257

**Published:** 2023-05-03

**Authors:** Justyna Fijolek, Elzbieta Radzikowska

**Affiliations:** The Third Department of Pneumonology and Oncology, National Tuberculosis and Lung Diseases Research Institute, Warsaw, Poland

**Keywords:** eosinophils, lymphocytes, inflammatory disorders, granulomatous inflammation, blood vessels

## Abstract

Eosinophilic granulomatosis with polyangiitis (EGPA) is a rare disease characterized by eosinophil-rich granulomatous inflammation and necrotizing vasculitis, pre-dominantly affecting small-to-medium-sized vessels. It is categorized as a primary antineutrophil cytoplasmic antibody (ANCA)-associated vasculitides (AAVs) but also shares features of hypereosinophilic syndrome (HES); therefore, both vessel inflammation and eosinophilic infiltration are suggested to cause organ damage. This dual nature of the disease causes variable clinical presentation. As a result, careful differentiation from mimicking conditions is needed, especially from HES, given the overlapping clinical, radiologic, and histologic features, and biomarker profile. EGPA also remains a diagnostic challenge, in part because of asthma, which may pre-dominate for years, and often requires chronic corticosteroids (CS), which can mask other disease features. The pathogenesis is still not fully understood, however, the interaction between eosinophils and lymphocytes B and T seems to play an important role. Furthermore, the role of ANCA is not clear, and only up to 40% of patients are ANCA-positive. Moreover, two ANCA-dependent clinically and genetically distinct subgroups have been identified. However, a gold standard test for establishing a diagnosis is not available. In practice, the disease is mainly diagnosed based on the clinical symptoms and results of non-invasive tests. The unmet needs include uniform diagnostic criteria and biomarkers to help distinguish EGPA from HESs. Despite its rarity, notable progress has been made in understanding the disease and in its management. A better understanding of the pathophysiology has provided new insights into the pathogenesis and therapeutic targets, which are reflected in novel biological agents. However, there remains an ongoing reliance on corticosteroid therapy. Therefore, there is a significant need for more effective and better-tolerated steroid-sparing treatment schemes.

## 1. Introduction

Eosinophilic granulomatosis with polyangiitis (EGPA) is a rare disease characterized by late-onset asthma, blood and tissue eosinophilia, and small-to-medium vessel vasculitis (1). It was first described in 1951 by two pathologists (J. Churg and L. Strauss), based on an analysis of autopsies of 13 patients with asthma, eosinophilia, and specific organ lesions, such as cardiac insufficiency, renal failure, and peripheral neuropathy (2). Its annual incidence and pre-valence range from 1 to 3 per 1,000,000 and 11 to 45 per 1,000,000, respectively, without gender dominance (3). However, the disease may be underdiagnosed because of restrictive pathomorphological criteria (2). Patients with asthma are a particular risk group, as they experience EGPA 34 times more frequently than those in the general population (4). The mean age at disease onset is approximately 50 years (5), although the disease can also occur in children (6).

Eosinophilic granulomatosis with polyangiitis is often diagnosed in pneumonological departments, where patients are referred due to asthma and lung lesions in chest computed tomography (CT) scans. In a recent study, among 46 consecutive patients hospitalized in a respiratory center because of peripheral eosinophilia and respiratory/lung symptoms (from 2017 to 2019), EGPA was the most common cause of these conditions (45.6%) (7). According to the current nomenclature classification, EGPA belongs to the group of antineutrophil cytoplasmic antibody (ANCA)-associated vasculitides (AAVs), along with granulomatosis with polyangiitis (GPA) and microscopic polyangiitis (MPA) (8), however, it is clearly distinct from GPA to MPA (9, 10). This is a unique disease sharing features of vasculitis and hypereosinophilic syndrome (HES) (11). In addition, these two processes are responsible for the heterogeneous clinical symptoms and phenotypes. Therefore, diagnosis is challenging and requires careful differentiation under mimicking conditions. ANCA are present less frequently than GPA and MPA (up to 30–40% of patients), and primarily target myeloperoxidase (MPO) (9, 10).

Given its rarity and unique features (such as eosinophilia and eosinophilic inflammation), EGPA has often been excluded from AAV studies, which has resulted in a delay in progress in knowledge about the disease compared to other AAVs. However, recently, increasing interest in EGPA as a subject of clinical trials has been observed, and new international projects concerning EGPA are being developed (12). Significant improvements in our understanding of the disease reflect meaningful progress in its early diagnosis and treatment. In this article, we discuss advances in EGPA, including its pathogenesis, diagnosis, and treatment, considering novel drugs that have or are being evaluated to improve patient outcomes.

Eosinophilic granulomatosis with polyangiitis has been defined mainly based on the histologic findings known since the first EGPA description by Churg and Strauss (2). According to the 1994 Chapel Hill Consensus Conference (CHCC), EGPA is defined as an eosinophil-rich and necrotizing granulomatous inflammation often involving the respiratory tract, with necrotizing vasculitis affecting small to medium vessels, and is associated with asthma and eosinophilia (13). In 2012, the nomenclature and classification system was revised. The former name “Churg-Strauss syndrome” was replaced with EGPA, and the disease was classified into a new group “ANCA-AAVs” alongside GPA and MPA (8). However, recent data indicate that the current terminology “EGPA” is not entirely appropriate and requires revision. Although it implies that EGPA is a genuine vasculitis *(“polyangiitis”)*, symptoms of vasculitis are not present in all patients, and it is still debated whether patients having asthma, hypereosinophilia, and eosinophil-rich granulomatous inflammation without necrotizing vasculitis, should be determined as having EGPA (14).

## 2. Pathogenesis and triggering factors

While the triggering factors for EGPA remain unknown, our understanding of its pathogenesis has significantly improved. The disease is considered an immune-inflammatory disorder based on the profound immunological dysregulation of both the innate and adaptive immune systems, including T and B lymphocytes, eosinophils, and neutrophils. In addition, genetic pre-dispositions have been reported (15).

### 2.1. T lymphocytes

In EGPA both Th1 and Th2 pathways are activated, and eosinophils contribute to organ damage (16). EGPA is mainly considered a Th2-response disease. This is evidenced by elevated serum levels of Th2-related cytokines (17, 18) and increased expression of Th2 and regulatory-type transcripts in bronchoalveolar lavage fluid (BALF) cells from patients with active EGPA (19). The T-cell receptors of patients with EGPA show a restricted repertoire (20), suggesting that an antigen-mediated process is likely responsible for their activation (1). Activated Th2 lymphocytes secrete many eosinophilotropic cytokines, including interleukins (IL) 3, 4, 5, 10, and 13, which enhance eosinophil maturation in the bone marrow and their peripheral activation (18, 19). Among these interleukins (ILs), IL-5 is the key cytokine that mediates the release of eosinophils into the bloodstream. It enhances eosinophil production, maturation, and activation and prolongs survival, mainly by inhibiting apoptosis (21), however, it is not responsible for fostering eosinophil infiltration of specific tissues (21). The relevance of the Th2 pathway is underlined by the efficacy of treatment based on the blocking of IL-5. IL-5 receptor (IL-5R) expression is specific to eosinophil differentiation, as it is almost exclusively expressed in eosinophils (22). Targeting IL-5 or IL-5R has become an attractive approach to treating eosinophil-related disorders, including EGPA (23).

Although the Th2 response plays a crucial role, Th17 and Th1 lymphocytes are also involved in EGPA pathogenesis. Th17 cells are specific lymphocytes that produce several proinflammatory cytokines (IL-17A, IL-17F, or IL-22) and are regulated by regulatory T-lymphocytes (Treg), which suppress the immune response and have a protective role in the development of autoimmune disorders (24). Elevated numbers of Th17 cells and decreased frequency of Treg cells have been found in patients with EGPA; the Th17/Treg ratio correlates well with markers of disease activity, and CCR4-active chemokines contribute to eosinophilia (25). The involvement of the Th1 pathway is evidenced by the increased serum concentration of interferon-gamma (IFN-γ) in EGPA patients (26). This cytokine is involved in granuloma formation to protect against the cytotoxic effects of eosinophils. Moreover, Th1 cells were detected in skin lesion biopsies (27), and the gut mucosa of patients with EGPA; the latter has a positive correlation with disease activity (28). Clonally expanded CD8 + T cells have also been described in patients with EGPA, suggesting their pathogenic role in vascular damage (29).

### 2.2. Eosinophils

Evidence supports that eosinophils play a key role in the pathogenesis of EGPA, with abnormal proliferation, impaired apoptosis, and increased tissue toxicity attributed to eosinophil products (5). Their increased number and extracellular protein deposition have been observed in various tissue specimens, including skin (30) and endomyocardial samples (31). The direct toxic effect is associated with the release of cytoplasmic granules upon eosinophil activation (32, 33). However, it can also be an indirect toxic effect as a result of the recruitment and activation of other inflammatory cells (26). There were two types of granule-characterized eosinophils. The primary granule contains Charcot-Leyden crystal proteins and lipid bodies, which are complex inducible organelles that are the site of eicosanoid synthesis, while the secondary granule contains a variety of pre-formed proinflammatory cytokines, enzymes, and growth factors, as well as specific cationic proteins [major basic protein (MBP); eosinophilic cationic protein (ECP); eosinophil peroxidase (EPO); eosinophil-derived neurotoxin (EDN)], which are mainly responsible for specific organ damage (26, 34). The effect of eosinophils depends largely on the tissue involved, however, complications of their accumulation and activation include thrombosis (34, 35), fibrosis (36), and allergic inflammation (26, 34, 37). In addition to being activated, eosinophils secrete many cytokines which enhance the Th2 response, thereby maintaining a vicious circle. Eosinophils are a key source of IL-25. Its elevated concentrations have been found in patients with EGPA and are associated with disease activity and the degree of eosinophilia (38).

In addition to the Th-2 pathway, eotaxins (CCL11-eotaxin, CCL24-eotaxin 2, and CCL26-eotaxin 3) are potent eosinophil activators. They are eosinophil-selected chemokines mainly secreted by endothelial cells but also by T lymphocytes; for example, both IL-4 and 13 released by Th2 cells are synergic promoters of eotaxin synthesis (39). Furthermore, eotaxin 3 is a particularly potent chemoattractant that binds to a specific CCR3 receptor (highly expressed in eosinophils) (22). Increased levels of eotaxin 3 have been described in patients with EGPA and are correlated with disease activity (40, 41).

One case report of Fip1-like1-plateled-derived factor receptor A (FIP1L1-PDGFRA) – positive EGPA implicated the role of tyrosine kinase pathways as drivers for eosinophilia in EGPA (42). The efficacy of imatinib in FIP1L1-PDGFR A-unmutated EGPA has also been previously described (43, 44). These findings indicate a possible shared pathogenic mechanism of EGPA with HES.

### 2.3. The innate immune system

Increased IL-33, thymic stromal lymphopoietin (TSLP), and type 2 innate lymphoid cells (ILC2) have been found in patients with active EGPA, indicating that the pathogenesis of EGPA involves interactions between the innate and adaptive immune systems (45). TSLP is a critical mediator of the Th2 response, acting on multiple cell lineages, including eosinophils and ILC2, affecting their maturation, survival, and recruitment. One activator of TSLP is IL-4, which is significantly increased in patients with EGPA. ILC2 are characterized by high expression of transcription factor 3 (GATA3) and production of IL-5 and IL-13 (28), which are key factors involved in the recruitment of eosinophils.

### 2.4. B lymphocytes, ANCA, and neutrophils

The role of B lymphocytes in the pathogenesis of EGPA has also recently been highlighted, although not well established, however, the promising results of anti-CD 20 B-cells depleting therapy can support this idea (46). In addition, many patients exhibit an abnormal humoral response, reflecting B lymphocyte activation. Elevated serum concentrations of total immunoglobulin E (IgE) and IgE-containing immune complexes are often observed in patients with EGPA (26). It has also been reported that immunoglobulin G subclass 4 (IgG4) levels are essentially increased (47) and correlated with the number of affected organs and disease severity in EGPA (48). Tsurikisawa et al. (49) showed a significant increase in the proportion of B lymphocytes positive for CD80, CD27, and CD95 in the blood of EGPA patients with frequent relapses, while those with the seldom-relapsing disease had higher CD19-positive B-cell counts and higher serum IgG levels, suggesting that frequently relapsing EGPA is associated with induced B-cell apoptosis. Finally, a comparison of lymphocyte immunophenotypes in EGPA patients showed that, in addition to increased T lymphocyte activity, they correlated with increased plasmablasts and T follicular helper lymphocytes (Tfh), indicating that B-cell activation is involved in the development of EGPA (50).

The presence of ANCA also reflects the activation of B lymphocytes, however, the pathogenic role of these antibodies in EGPA has not been firmly established and is suspected to be similar to MPA. Animal models have shown that MPO-ANCA has a direct damaging effect on endothelial cells, resulting in the development of necrotizing crescentic glomerulonephritis and pulmonary hemorrhage (51). In a human case study, a newborn was reported to develop pulmonary-renal syndrome with the placental transmission of MPO-ANCA (52). A study conducted by Falk et al. (53) made a breakthrough regarding the pathogenic role of ANCA in AAVs. The study proved that ANCA can activate primed neutrophils to produce reactive oxygen species (ROS) and release lytic enzymes that cause necrosis of endothelial cells and adjacent matrix. Unlike GPA and MPA, where the role of ANCA is well established, in EGPA it is still not fully understood. First, ANCA is detected in only one-third of patients, less frequently than GPA and MPA (9, 54, 55). Second, although EGPA and MPA are characterized by the same type of ANCA (anti-MPO), the diseases differ significantly in their clinical phenotype [e.g., renal involvement or diffuse alveolar hemorrhage (DAH) is much more frequent and more severe in MPA than in EGPA] (56). It has been suggested that alternative MPO epitopes, other than those in MPA, develop in ANCA-positive EGPA, contributing to mitigated vascular features (15). Finally, the presence of ANCA in EGPA does not always correlate with symptoms of vasculitis (14). Some authors speculate that for EGPA, a different targeted epitope, a change to the specific epitope conformation, or a failure in the masking process of this epitope by the ceruloplasmin fragment could explain the presence of MPO-ANCA (57).

In recent years, there has been much interest in neutrophil extracellular traps (NETs). NETs are defined as a network of chromatin threads containing histones and proteolytic enzymes (including MPO) that can be released by activated neutrophils to kill bacteria (58). Furthermore, NETs are considered to play an important role in the pathogenesis of AAVs and are a source of ANCA (59, 60). However, a recent study demonstrated enhanced NETs in patients with EGPA with no regard to ANCA status, significantly correlated with blood eosinophil count (61). Eosinophil extracellular traps (EETs) and eosinophil ETosis (EETosis) have also recently been studied in EGPA (62). Mukherjee et al. (63) demonstrated that immunoprecipitated immunoglobulins from ANCA (+) sputum derived from patients with EGPA allowed extensive EETs from both neutrophils and eosinophils *in vitro*. Direct evidence of EETs/EEtosis within the thrombus in patients with EGPA has been also provided (64).

### 2.5. Genetics

Several immunogenetic factors that pre-dispose patients to EGPA have been identified. It has been shown that the HLA-DRB1*07 and DRB1*04 alleles are associated with the development of EGPA, while DRB1*03 and DRB1*13 are protective (65). Another genetic risk factor is HLA-DRB4, which suggests a strong link with CD4 + T lymphocyte activation (66). In turn, functionally relevant variations in the IL-10 gene promoter (IL-10.2 haplotype) are associated with ANCA-negative EGPA (67).

Recently, a genome-wide association study (GWAS) demonstrated that ANCA status in EGPA is associated with a specific genetic background (56). EGPA with ANCA positivity is associated with human leukocyte antigen DQ (HLA-DQ), which shares both clinical and major histocompatibility complex (MHC) associations with anti-MPO AAV. In turn, ANCA-negative EGPA has a mucosal barrier origin and is associated with variants of the glycoprotein A33 (GPA33) and IL-5/interferon regulatory factor 1 (IRF1) (genotype sharing with asthma). There was an association of both EGPA subgroups (ANCA + and ANCA –) with variants at the TSLP, BCL2L11, and CDK6 loci and suggestive evidence for BACH2, Chromosome 10, and lipoma preferred partner (LPP), indicating that EGPA is characterized by certain genetic variants associated with the syndrome as a whole (56).

## 3. Triggering factors

There are no well-known triggering factors of EGPA, however, environmental factors, infections, and drugs have been speculated. Several cases of disease development following massive antigen inhalation (grain dust, flour dust, and cereal dust) (68) and exposure to pigeons have been described (69). Regarding infectious agents, *Aspergillus fumigatus* triggers EGPA. Some reports demonstrated that *Aspergillus* might be a pathogen common to both allergic bronchopulmonary aspergillosis (ABPA) and EGPA, and prolonged exposure to this fungus in some patients with ABPA may promote progression to EGPA (70). A case of concomitant ABPA and EGPA after *Aspergillus niger* infection has also been reported (71). Other infectious agents include viruses, among others. A case of EGPA following COVID-19 has been recently reported (72).

Other factors include drugs mainly used in asthma, such as leukotriene receptor antagonists (LTRAs) or anti-IgE antibodies, which are also suspected to induce EGPA (73, 74), however, the mechanism to induce vasculitis is not well-known. One hypothesis is that the administration of these drugs in the asthmatic phase of undiagnosed patients with EGPA may result in vasculitis burst due to reducing the steroid dose, previously masking symptoms of EGPA (5). Two case-controlled studies concluded that treatment with LTRAs did not increase the risk of EGPA (4, 75). However, a recent monocentric retrospective study found a significant correlation between LTRAs exposure and ANCA positivity in EGPA patients. The authors speculated that LTRAs could induce imbalanced stimulation of leukotriene receptors, which may cause neutrophil activation, NETs production, and subsequent ANCA stimulation, resulting in the development of vasculitis (76). Other suspected drugs include anti-IL therapies. Ikeda et al. (77) described a case of EGPA that became apparent following the discontinuation of dupilumab (anti-IL-4/IL-13 antibody). Additionally, Lim et al. (78) reported a case of EGPA during benralizumab (anti-IL5Rα) treatment.

As asthma is a major feature, allergy may also contribute to the development of EGPA. However, systematic allergy testing in patients with EGPA revealed evidence of allergy in less than one-third of patients (79). Other suspected factors include vaccination and desensitization (5). A case of EGPA that developed following a booster dose of the anti-SARS-CoV-2 vaccine has also been reported (80).

## 4. Clinical symptoms and disease stages

Classically, EGPA develops in three consecutive stages. The first is the prodromal phase dominated by asthma and allergic rhinosinusitis. After a variable period (mean 9.3 ± 10.8 years) (5), the eosinophilic phase develops—characterized by peripheral and tissue eosinophilia, which may result in pulmonary infiltrates, eosinophilic cardiomyopathy, or gastrointestinal involvement (GI). Next, the disease progresses into the vasculitic phase, in which organ manifestations consistent with vasculitis pre-dominate (81). However, disease succession does not always occur. In some patients, there is an overlap of these phases, or the disease may begin with the eosinophilic phase; in others, the absence of either eosinophilic or vasculitic phases is observed (1, 34). This complexity of the disease makes the clinical manifestation diverse. Interestingly, the spectrum of manifestations varies depending on the patient recruitment center, e.g., patients admitted to respiratory departments have more frequent cardiac involvement and limited features of vasculitis (14). The frequencies of organ involvement and phenotypic features in the selected EGPA cohort are presented in Table 1.

**TABLE 1:** Organ involvement and phenotypic features of selected eosinophilic granulomatosis with polyangiitis (EGPA) cohorts.

	Guillevin et al. (99)	Comarmond et al. (55)	Durel et al. (100)	Sinico et al. (9)	Moosig et al. (82)	Tsurikisawa et al. (83)	Samson et al. (54)	Saku et al. (101)	Durel et al. (134)	Healy et al. (85)	Bettiol et al. (86)	Fijolek et al. (87)
No. of pts	96	383	101	93	150	121	118	188	63	93	573	86
M/F (n)	44/52	199/184	43/58	39/54	76/74	42/79	64/54	121/67	27/36	ND	276/297	35/51
Country	France	France	France, Italy, UK	Italy	Germany	Japan	France, Belgium, UK	Japan	France, Italy, Belgium, UK	New Zealand USA	Italy, Austria, UK	Poland
Study period	1963–1995	1957–2009	1990–2011	1989–2004	1990–2009	1999–2015	2005–2011	1996–2015	1990–2011	1997–2003	1988–2018	1992–2020
Center	Internal Medicine	FVSG	Internal Medicine, Allergology, Immunology	Nephrology, Immunology, Rheumatology, Pulmonology, Neurology	Rheumatology, Internal Medicine, Otorhinolaryngology, Ophthalmology, Cardiology	Allergology, Respirology	Internal Medicine, Immunology, Allergology, Pulmonology	Internal Medicine, Immunology, Rheumatology	Nephrology, Internal Medicine	Internal Medicine, Allergology, Immunology	Internal Medicine, Surgery, Rheumatology, Allergology, Pulmonology, Nephrology	Pulmonology
Age at onset of EGPA (mean or median; yrs)	48.2	50.3	49.2	51.6	49.1	53.3	51.9	59.7	60 (median)	ND	55.3 (median)	35 (median)
Eosinophil count/m^3^ (mean or median)	7,193	7,569	ND	4,400	1,100	8,528	8,231	8,775	3,650 (median)	ND	2,680	5,000 (median)
ANCA (+) (%)	47.6	31.0	42.6	37.0	30.0	35.0	41.0	47.0	84.0	16.1	50.1	14.0
Asthma (%)	100.0	91.1	100.0	95.7	92.7	98.3	94.0	95.2	100.0	100	96.3 (lower respiratory tract)	96.5
Sinusitis (%)	61.1	41.8	92.1	77.4	76.7	91.2	68.0	50.0	70.0	63.4	79.4 (ENT)	82.6
**Organ manifestation (%)**												
Lungs	37.5	91.4	54.5	50	61	67.6	98	34.6	38.0	65.6	ND	88.4
Nerve	78.1	55.1	66.3	64.5	76	98.3	74	88.3	46.0	52.0	63.2	54.6
Heart	13.5	27.4	20.8	16.1	46	73.9	38	11.2	14.0	28.0	21.3	76.7
GI	33.3	23.2	25.0	21.5	28	78.6	29	12.2	ND	17.2	10.1	19.8
Skin	51.0	39.7	46.5	52.7	49	67.9	48	41.5	40.0	67.7	36.6	43.0
Kidneys	26.0	21.75	26.0	26.9	18	35.2	27	18.1	86.0	17.2	13.8	16.3

ANCA, antineutrophil cytoplasmic antibodies; GI, gastrointestinal involvement; ENT, ear, nose, throat; FVSG, French Vasculitis Study Group; anti-MPO, antimyeloperoxidase; anti-PR3, antiproteinase 3; ND, no data.

### 4.1. The prodromal phase

Asthma is a major feature of EGPA usually preceding the symptoms of vasculitis (mean 9.3 ± 10.8 years) (5). It concerns 90–100% of patients (14, 54, 82–87) and is characterized by distinct features compared to asthmatic patients in the general population. First, it is usually late-onset asthma, which begins in adulthood at around 30–40 years of age. Second, an allergic background is present in less than one-third of patients with EGPA, compared with approximately 70% of patients with asthma in general, and there are no seasonal exacerbations (79). Atopy, if present, is associated with a better prognosis but with more severe or uncontrolled asthma manifestations in the year before the development of vasculitis (88). Third, asthma in EGPA is usually severe and often requires long-term treatment with oral corticosteroids (CS) despite the regression of systemic disease. In a retrospective study of 157 patients with EGPA, asthma was severe in 57% of cases, whereas persistent airflow obstruction was present in 38, 30, and 46% of patients at diagnosis, 3-year follow-up, and final visit, respectively (89). In another study, airflow obstruction was observed in approximately 40% of patients in clinical remission (90). It remains unclear why systemic therapy controls systemic manifestations in EGPA, but not asthma symptoms. Some authors speculate a dissociation between eosinophil bone marrow production and eosinophil recruitments in the airways which results that in sputum (but not blood), eosinophilia is still present in the group of EGPA patients in remission phase (91). Asthma, although often severe, may paradoxically improve during the full-blown vasculitic phase (92). However, it has recently been demonstrated that the severity of asthma increases 3–6 months before the onset of systemic symptoms (89). Furthermore, severe or uncontrolled asthma is associated with baseline pulmonary and ear, nose, and throat (ENT) manifestations but not with clear-cut vasculitic features (93).

Finally, asthma in EGPA is often accompanied by allergic manifestations in the upper respiratory tract, such as allergic rhinitis, chronic sinusitis (70–90%) (89), and nasal polyps (42–58%) (89, 94–96). At this stage of the disease, distinguishing prodromal ENT symptoms in the course of EGPA from chronic rhinosinusitis with nasal polyps (CRSwNP) is challenging; especially in the biopsy, both typical histological features of eosinophilic polyposis are present (96, 97). Lesions observed in GPA, such as destructive granulomatous inflammation or nasal crusting, are uncommon in EGPA. However, secretive otitis media, chronic ear drainage, sensorineural hearing loss, and facial nerve paralysis may occur (34, 98).

### 4.2. The eosinophilic phase

In this phase, clinical symptoms are due to eosinophilic infiltration of organs. Typically, the lungs, gastrointestinal tract, and heart are affected.

Lung involvement is present in 37–98% of patients with EGPA, depending on the study series (9, 55, 84, 99–102). In addition, a chest radiograph is abnormal in 70% of patients and shows bilateral pulmonary consolidative or reticulonodular opacities in a peripheral distribution (103). In high-resolution computed tomography (HRCT), which is a more precise method, pulmonary lesions can be classified as airspace and airway patterns (104), however, both types often coexist in one patient. Furthermore, all lung imaging changes observed in EGPA are not EGPA-specific and are frequently observed in other diseases (7, 105). The airspace pattern is mostly migrating patchy infiltrates with peripheral dominance corresponding to chronic eosinophilic pneumonia (EP), (104, 106) which antedate systemic vasculitis in 40% of cases (81). Other common findings are ground-glass opacities (39–53%), followed by consolidations (28–42%), and poorly defined nodules (24–63%) (89, 106). The airway pattern consists of small centrilobular nodules, tree-in-bud sign, bronchial dilatation, wall thickening, and mosaic perfusion pattern (89, 104, 106), which reflect airway involvement in the course of asthma generally, not only in EGPA (7). Greater severity and longer duration of asthma (>5 years) are significantly associated with a higher incidence of airway abnormalities on HRCT in patients with EGPA (107). Histologically, small nodules correspond to eosinophilic bronchiolitis and peribronchiolar vasculitis, whereas bronchial wall thickening is associated with airway wall eosinophil and lymphocyte infiltrations (106).

Other less frequent thoracic symptoms of EGPA include pleural effusion and hilar or mediastinal lymphadenopathy (108, 109). Pleural effusion may develop secondary to eosinophilic pleurisy as well as eosinophilic cardiomyopathy-associated congestive heart failure (1). Other HRCT findings may include interstitial edema, cardiac enlargement, or pericardial effusion, all of which are related to cardiac involvement. In some patients, these HRCT findings may be the only chest symptoms.

A small proportion of patients (3–4%) may experience DAH, which is a life-threatening vasculitic manifestation that can lead to acute respiratory distress (16).

GI is less common in EGPA, although it is significantly more frequent than in GPA or MPA (84). This organ manifestation is recognized in 24–78% of patients, depending on the series and diagnostic tests used (54, 55, 82–84, 100). Manifestations are non-specific and include abdominal pain, which is the most frequently reported symptom (30–91%) (54, 100, 110), followed by diarrhea (45%) (110) and minor bleeding (3–9%) (54, 82, 100). Cholecystitis, pancreatitis, intestinal infarction, and ischemic colitis have been described, but they are rarely present (1–3%) (54, 102). In a study of 383 patients with EGPA, symptoms of acute surgical abdomen occurred in approximately 6% of the cases (55). In another study, 22–45% experienced severe GI manifestations, potentially requiring surgery (16). In EGPA, clinical GI symptoms and findings on abdominal CT are non-specific and require differentiation from other diseases. Common CT features include bowel enlargement and pathologic enhancement (16), whereas histological examination demonstrates mainly eosinophilic infiltrations, sometimes with vasculitis and eosinophilic granulomas (5, 102, 111).

Among the three types of AAVs, cardiac involvement (CI) is most common in EGPA and is mostly present in ANCA-negative patients (9, 10, 55, 112). In Churg and Straus’s original cohort, it occurred in more than 50% of autopsies (2), however, its reported incidence varies from 11 to 74%, depending on the series and diagnostic techniques used (54, 55, 82, 84, 101, 113, 114). Clinical manifestations are variable and include myocarditis (often with thrombus formation), pericarditis, valvular insufficiency, or involvement of the conduction system, resulting in arrhythmia (5, 98, 115–117). The severity of clinical symptoms varies from mild to clinically overt and life-threatening. Patients most often complain of chest pain and dyspnea (116, 118, 119), but the first symptom may also be acute congestive heart failure, life-threatening arrhythmia, and cardiac death (119). In addition, cardiac involvement can be asymptomatic (83, 118, 120–123). In recent data of Polish 86 patients with EGPA, cardiac invasion was found in 76.7% of the cases, with almost 30% of the cases being asymptomatic (87).

Eosinophilia and its cytotoxicity play a crucial role in heart damage caused by EGPA (119). Patients with CI have been reported to have significantly higher eosinophil counts at diagnosis than those without this organ manifestation (118, 124); usually, they were younger, had negative ANCA, higher disease activity, and higher C-reactive protein (CRP) levels (118). Three successive stages of eosinophilic cardiac damage have been described. The first stage is necrosis due to the infiltration of eosinophils and the release of granular proteins. The second phase is characterized by thrombosis formation, whereas fibrosis of the endocardium and valves occurs in the final stage, resulting in restrictive cardiomyopathy and cardiac insufficiency (119). This phase corresponds to scarring of the endomyocardium and is irreversible; therefore, early detection of cardiac involvement is crucial for prognosis. This is because treatment at the earlier stages provides a chance to reverse the inflammatory process and limit myocardial necrosis.

CI of EGPA can also be derived from coronary vasculitis, which is a rare situation occurring in approximately 3% of patients and manifests as myocardial infarction with negative results on coronary angiography (82, 113).

### 4.3. The vasculitic phase

This phase manifests as a feature of vasculitis. Typically, the nervous system, skin, and kidneys are affected, with the latter being the rarest. However, every organ may be involved. This phase is often preceded by general symptoms such as fever, weakness, muscle pain, or arthritis.

Involvement of the nervous system is a prominent feature of the vasculitic phase. It affects 42–76% of EGPA patients (54, 55, 82, 84, 87, 102), mainly ANCA-positive (9, 10). Among other forms of AAVs, it is most prevalent in EGPA (65 vs. 23% in MPA, and 19% in GPA) (125). Frequently affected nerves include the peroneal, tibial, ulnar, and median nerves, but the typical presentation is mononeuritis multiplex, usually manifested by foot drop and symmetrical polyneuropathy, often progressing when left untreated (126). Patients complain of numbness, burning sensation, pain, limb weakness, and other sensory disturbances, which can be the first symptom, even in 63% of the cases (126, 127). Diagnosis is mainly based on clinical evaluation and may be confirmed by electromyography (EMG) or nerve biopsy. However, the latter procedure is infrequently performed in clinical practice. In a large study of 955 AAV patients, only 12% underwent nerve biopsies, of which 53% had definitive vasculitis (125). Pathophysiologically, nerve damage is caused by vasculitis and eosinophilic infiltrates, with the latter pre-dominating in ANCA-negative cases (128).

Central nervous system (CNS) involvement in EGPA is less common and is reported in 5–29% of cases with neurological symptoms (54, 55, 82, 83). The main neurological manifestations included ischemic cerebrovascular lesions (52%), intracerebral and/or subarachnoid hemorrhage (24%), loss of visual acuity (33%), and cranial nerve palsies (21%). The clinical course varies, with long-term neurological sequelae being common (43%). Intracerebral hemorrhages have the worst prognostic impact (129).

Skin involvement is the next most prominent feature of the vascular phase. Its frequency ranges from 23 to 68% in patients (54, 55, 83, 84, 87, 100, 101, 113), with vascular purpura being the most common (24–39%) (54, 55, 100, 113). Other findings include subcutaneous nodules that occur in 30% of cases (5) and less frequently, non-specific maculopapular rash, urticaria, petechiae, sterile pustules, livedo reticularis, vesicles, and pruritus (130). A wide range of histological changes is observed in the purpura of the skin, from eosinophilic vasculitis to leukocytoclastic vasculitis without eosinophilic infiltration, making diagnosis difficult (131). Other skin lesions in EGPA show extensive infiltration of eosinophils and surrounding inflamed small dermal blood vessels (132), however, eosinophil infiltration is not specific to EGPA and is a common finding in a broad spectrum of skin diseases (133).

In EGPA, renal involvement is less frequent and less severe than in other forms of AAV (134). In addition, its reported frequency depends on the profile of the medical facility. According to various studies from different centers, the frequency varies from 16.3 to 35% of patients (54, 55, 82–84, 87, 100, 113), with nephrological facilities even in 86% of patients presenting with renal diseases at vasculitis diagnosis (134). Renal involvement in EGPA pre-dominates in ANCA-positive patients, which is in line with the aforementioned study, in which 84% of patients had a positive ANCA test (134). The most common clinical symptom reported in different series was proteinuria (3.3–20%) (55, 82, 113), with renal insufficiency observed in 4.3–15% of cases (55, 83), and up to 75% of patients referred to nephrological facilities, in whom acute renal failure was the most common renal presentation (134). Histologically, the most typical pattern included pauci-immune necrotizing glomerulonephritis (78%), followed by membranous nephropathy (10%) and membranoproliferative glomerulonephritis (3%), both of which were ANCA-negative. Other findings include pure acute interstitial nephritis (10%) and interstitial eosinophilic inflammation in half of the patients, regardless of ANCA status (134).

## 5. Diagnosis, classification, and disease phenotypes

The diagnosis of EGPA is challenging and requires the correlation of clinical, laboratory, radiologic, and histopathologic findings, however, in cases with a history of asthma, eosinophilia, and both “vasculitic” and “eosinophilic” organ damage, the suspicion of EGPA is quite straightforward - in contrast to those with incomplete manifestations, which can be difficult to recognize. In addition, some patients lack evidence of vasculitis or ANCA, and there is an ongoing debate over whether EGPA can be recognized in these cases. Histology can confirm the diagnosis of EGPA, but the simultaneous presence of all three typical lesions is rare (135). In clinical practice, the diagnosis of EGPA is mainly clinical, however, considering the rarity of the disease and the variety of symptoms, the accuracy of the diagnosis increases with a multidisciplinary discussion among experienced clinicians (16, 103).

### 5.1. Diagnostic and classification criteria

To date, there are no validated or universally accepted diagnostic criteria for EGPA. The aforementioned CHCC is a nomenclature classification and not a diagnostic classification (8, 13). The first diagnostic criteria were proposed by Lanham et al. (81) which included asthma, eosinophilia ≥ 1,500 cells/μL, and manifestations of vasculitis involving at least ≥2 extrapulmonary organs. These criteria were developed before classifying EGPA into AAVs and do not require histological examination. However, they have been widely used by clinicians owing to their simplicity in capturing the essence of the disease. Recently, the Joint Task Force of the European Respiratory Society (ERS) and the Foundation for the Development of Internal Medicine in Europe (Groupe d’Etudes et de Recherche sur les Maladies Orphelines Pulmonaires; GERM’O’P) proposed new diagnostic criteria (14, 136). They restricted the EGPA terminology to ANCA-positive cases and/or to those with genuine features of vasculitis (or with surrogates of vasculitis) that are precisely defined. In addition, they proposed that patients with asthma, blood eosinophilia, and systemic manifestations, but non-vasculitic and without ANCA, are referred to as having hypereosinophilic asthma with systemic manifestations (HASM), not EGPA. The next criteria are those used in the MIRRA study assessing the safety and efficacy of mepolizumab in patients with EGPA (23). In contrast to the above-mentioned criteria, they were very loose, with the majority of patients not having ANCA or features of vasculitis. However, these criteria were developed for the purposes of a clinical trial (as an eligibility criteria), and are not widely used in clinical practice.

Classification criteria are often mistakenly used as diagnostic criteria, although they are not. Classification criteria were designed to distinguish EGPA from other types of vasculitis; therefore, they should be used only when a diagnosis of small- or medium-sized vessel vasculitis has been established.

The first classification criteria for EGPA were published in 1990 by the American College of Rheumatology (ACR). They were developed by comparing 20 EGPA-diagnosed patients with 787 control patients with other forms of vasculitis and included six items: asthma, eosinophilia > 10%, neuropathy, pulmonary infiltrates, sinusitis, and extravascular eosinophils in the biopsy. The presence of ≥4 of these six criteria allowed the classification of vasculitis as EGPA (137). These criteria were characterized by low sensitivity (67.1%, with 17% of cases meeting the criteria for other vasculitides), and although the specificity was high (64–98.9%), up to 27% of the comparators fulfilled at least one of these criteria (12). Despite poor methodology and lack of validation, these criteria have remained unchanged for several decades. In 2022, the ACR/EAAR (European Alliance of Associations for Rheumatology) established new classification criteria based on a prospective international multisite observational study (Diagnostic and Classification Criteria in Vasculitis; DCVAS project) conducted at 136 sites from 32 countries, including 107 cases of EGPA and 450 comparators. These criteria highlight the significance of peripheral eosinophilia, asthma, and eosinophilic inflammation and specify other features that function as important disease classifiers (such as mononeuritis multiplex, obstructive airway disease, or nasal polyps). Moreover, unlike the previous 1990 criteria, these are validated, have excellent sensitivity (85%) and specificity (99%), and incorporate ANCA testing. The criteria include seven items that have been assigned a point weight (positive or negative), and vasculitis could be classified as EGPA if the cumulative score was ≥6 points (138). Although these criteria were developed primarily for clinical trial purposes, they represent a major advancement in clinical practice as well, however, they are only for EGPA classification and do not solve the problem with diagnosis. A summary of the proposed diagnostic and classification criteria for EGPA (including the definition of the disease) is presented in Table 2.

**TABLE 2:** Eosinophilic granulomatosis with polyangiitis – definition, diagnosis and classification.

Definition Chapel Hill 2012	Diagnostic criteria			Classification criteria	
	Lanham criteria (1984)	Criteria proposed by the ERS-task force and GERM’O’P (2013, 2017)	Criteria proposed in the MIRRA trial (2017)	ACR 1990	ACR 2022
Eosinophil-rich and necrotizing granulomatous inflammation often involving the respiratory tract, and necrotizing vasculitis pre-dominantly affecting small to medium vessels, and associated with asthma and eosinophilia; ANCA is more frequent when glomerulonephritis is present Nasal polyps are common Limited expression of EGPA confined to the upper or lower respiratory tract may occur Granulomatous or non-granulomatous extravascular inflammation, such as non-granulomatous eosinophil-rich inflammation of lungs, myocardium, and gastrointestinal tract is common	1. Asthma 2. Blood eosinophilia >1,500 cells/mm^3^ or >10% of WBC 3. Evidence of vasculitis involving two or more extrapulmonary organs (with or without biopsy) All 3 criteria must be meet	Asthma and eosinophilia >1,500 cells/mm^3^ 1. Definite vasculitis features, as: biopsy-proven necrotizing vasculitis of any organ, biopsy proven necrotizing glomerulonephritis or crescentic glomerulonephritis, DAH, palpable purpura, myocardial infarction due to proven coronaritis 2. Definite surrogates of vasculitis, as: hematuria associated with red casts or >10% dysmorphic erythrocytes or hematuria and 2 + proteinuria on urinalysis, or leukocytoclastic capillaritis and/or eosinophilic infiltration of the arterial wal lat biopsy 3. Mononeuritis or mononeuritis multiplex 4. ANCA and any systemic manifestation (extrapulmonary and non-ENT) At least one of the criteria is needed	A history or presence of asthma and eosinophilia >1,000 cells/mm^3^ or >10% of WBC 1. Histo-pathological evidence of eosinophilic vasculitis, perivascular eosinophilic infiltration, or eosinophil-rich granulomatous inflammation 2. Neuropathy 3. Pulmonary infiltrates 4. Sinonasal abnormality 5. Cardiomyopathy 6. Glomerulonephritis 7. DAH 8. Palpable purpura 9. ANCA positivity At least two of the criteria are needed	1. Asthma 2. Eosinophilia >10% of WBC 3. Neuropathy, mono- or polyneuropathy 4. Pulmonary infiltrates 5. Paranasal sinus abnormality 5. Extravascular eosinophils in biopsy The presence of any 4 or more of these 6 criteria are needed	1. Obstructive airway disease (+3) 2. Nasal polyps (+3) 3. Mononeuritis multiplex (+1) 4. Eosinophilia ≥1z10^9^/liter (+5) 5. Extravascular eosinophilic-pre-dominant inflammation on biopsy (+2) 6. Positive test for cANCA or anti-PR3 (−3) 7. Hematuria (−1) A score of ≥6 is needed

EGPA, eosinophilic granulomatosis with polyangiitis; ACR, American College of Rheumatology; ERS, European Respiratory Society; GERM’O’P, Groupe d’Etudes et de Recherche sur les Maladies Orphelines Pulmonaires; ANCA, antineutrophil cytoplasmic antibodies; DAH, diffuse alveolar hemorrhage; anti-PR3, anti-proteinase 3 antibodies; ENT, ear, nose, throat; WBC, white blood count.

It is worth noting that owing to its dual nature, EGPA has been also listed as an “associated syndrome” in the classification of HESs (11).

### 5.2. Diagnostic tests and differential diagnoses

To date, there are no reliable biomarkers of EGPA. The results of these studies were inconclusive, with varying success rates (Table 3) (17, 40, 45, 139–151). Active EGPA is characterized by marked eosinophilia, usually ≥1,500 cells/μL or >10%, which correlates with disease activity (1, 5). It is a fixed feature of EGPA and an important diagnostic criterion, however, in patients treated with systemic CS (e.g., asthma), eosinophil count may rapidly decline within a few days, and the results may be falsely normal (5). A significant proportion of patients have elevated inflammatory markers, such as C-reactive protein (CRP) and erythrocyte sedimentation rate (ESR), mainly at the onset of the disease (55). Non-specific elevations in IgE levels were detected in 75% of cases (26). MPO-ANCA should be tested with antigen-specific immunoassays in any patient with eosinophilic asthma and clinical features suggestive of EGPA (such as constitutional symptoms, purpura, polyneuropathy, unexplained heart, gastrointestinal or renal disease, and/or pulmonary infiltrates or hemorrhage) (152), however, only approximately one-third of patients are ANCA-positive (9). Recently, a novel observation of ANCA reactivity in the sputum of seronegative EGPA patients was reported (63). ANCA reactivity was associated with more severe respiratory symptoms and sputum eosinophilia. It is now being investigated whether ANCA sputum could be useful as a diagnostic tool for serum ANCA patients with EGPA as well as to identify a subset of patients with eosinophilic asthma who are at increased risk of developing EGPA in the future (63).

**TABLE 3:** Selected studies investigated biomarkers in EGPA.

Investigated biomarker	Patients’ cohort	Method	Results	Conclusion	References
Eotaxin-3	EGPA: 37 (15 active, 22 inactive), Healthy controls: 123 Disease controls: 138 (other AAV, HES, parasitic disease, SLE, SSc, CU other causes of eosinophilia).	Comparison of serum levels of eotaxin-3 in all groups, *ex vivo* stability of eotaxin-3 in serum samples testing, and determination of the association of SNPs in the eotaxin-3 gene.	1. Serum eotaxin-3 was highly elevated only in active EGPA (specificity: 87.5%, sensitivity: 98.6% at a cut-off level of 80 pg/ml). 2. None of the tested SNPs within the eotaxin-3 gene influenced the susceptibility to develop EGPA.	Serum eotaxin-3 is a sensitive and specific marker for the diagnosis of active EGPA. SNPs in the eotaxin-3 gene do not predict the risk of developing EGPA.	Zwerina et al. (41)
Eotaxin-1, Eotaxin-2, Eotaxin-3	EGPA: 40 (active) Healthy controls: 30 Disease controls: 57 (asthma, other AAV, HES)	Evaluation of serum eotaxin-1, 2, and 3 levels in all groups; identification of eotaxin-3 expression in tissue biopsies of EGPA.	Eotaxin-3 serum level was highly elevated only in active EGPA and correlated with blood eosinophil count, total IgE, and acute-phase parameters, with strong expression of eotaxin-3 in tissue biopsies of EGPA.	There is a significant association of eotaxin-3 with EGPA activity and blood eosinophil count.	Polzer et al. (40)
ECP	EGPA: 18 (11 active, 7 inactive) Healthy controls: 15	Serum levels of ECP evaluation in all groups.	Mean ECP serum level was significantly higher in active EGPA and correlated with blood eosinophil count.	ECP may be used as a disease activity marker in EGPA.	Guilpain et al. (139)
CCL17/TARC	EGPA: 25 (12 active, 13 inactive) HES: 18 Other AAV: 12 Other eosinophilia: 14 Healthy controls: 21	1. Serum levels of CCL17/TARC evaluation in all groups. 2. Identification of CCL17/TARC in tissue biopsies of EGPA.	1. Serum levels of CCL17/TARC were significantly elevated in active EGPA and correlated with blood eosinophil count, however, they are also noted in other eosinophilic diseases. 2. Expression of CCL17/TARC in the affected tissue of EGPA was found.	Serum levels of CCL17/TARC reflect EGPA activity. However, further studies to validate its use as an activity marker in EGPA are warranted.	Dallos et al. (140)
IgG4	EGPA: 46 (24 active, 22 inactive) GPA: 26 Atopic asthma: 25 Healthy controls: 20	Serum levels of IgG4 in all groups, assessment of tissue infiltration by IgG4 plasma cells	1. IgG4 levels were significantly higher in active EGPA and correlated with the number of disease manifestations and BVAS, and dropped during disease remission. 2. Tissue analysis did not show an increased IgG4 plasma cell infiltration.	Serum IgG4 levels are markedly elevated in active EGPA and correlate with the number of organ involvement and disease activity.	Vaglio et al. (48)
Blood eosinophil count, IgE, ESR, CRP	EGPA: 141 (mostly on treatment, during remission or mild disease activity; BVAS/WG = 1 or 2).	Parameters were measured quarterly (together 892 study visits)	1. Correlations between blood eosinophil count, IgE, ESR and CRP were mostly low or non-significant. 2. When BVAS/WG ≥1 defined active disease, the eosinophil blood count was weakly predictive of flare. 3. When BVAS/WG ≥3 defined active disease, ESR was weakly predictive of flare.	The blood eosinophil count, IgE, ESR and CRP have limitations as longitudinal biomarkers of disease activity or predictors of flare in EGPA.	Grayson et al. (141)
CCL17/TARC, eotaxin-3, IgG4, IgG4/IgG	EGPA: 25 (most patients on treatment with CS or IS, 18 disease flares during study period)	Evaluation of serum CCL17/TARC, eotaxin-3, IgG4 levels and IgG4/IgG ratio at each visit (together 105 study visits)	1. None of the biomarkers were useful to discriminate between active disease and remission. 2. Patients treated with CS had lower eotaxin-3 and blood eosinophil count levels compared to those not taking CS, irrespective of disease activity. 3. Use of IS was not associated with biomarkers levels.	Serum levels of CCL17/TARC, eotaxin-3, IgG4, and IgG4/IgG ratio do not clearly differentiate active and inactive EGPA.	Dejaco et al. (17)
Periostin	EGPA: 49 (46 had active disease within the past 28 days, 3 had active disease since the prior visit).	Evaluation of serum periostin levels at each visit (together 186 study visits)	1. No association between periostin level and presence or absence of disease flare was found. 2. An increase in periostin level was significantly associated with greater disease severity during a flare. 3. Periostin levels in EGPA were significantly higher than previously studied healthy controls and patients with asthma.	In EGPA, serum periostin level is modestly associated with greater disease severity during a flare, however, it does not discriminate active from inactive disease.	Rhee et al. (142)
A panel of 54 cytokines and chemokines	EGPA: 50 (40 active, 10 inactive) HES: 6 Asthma: 8 Healthy controls: 10	Evaluation of 54 cytokines and chemokines in the sera of all group, results were compared between disease and control groups.	1. Significant differences were only observed in serum levels of MDC, IL-8, MIP-1α and 1ß, and TNF-α, each of which were lower in active EGPA than in healthy controls, and differences between active EGPA and other disease groups did not reach significance. 2. Comparison between sera from active or inactive EGPA were not significance for any of the studied cytokines/chemokines.	No clear difference in the serum levels of measured cytokines and chemokines helped distinguish between active or inactive EGPA, or other disease or control groups.	Pagnoux et al. (143)
Anti-alpha-enolase antibodies	EGPA: 33 (24 active, 9 inactive)	Evaluation of anti-alpha-enolase antibodies, ANCA, ANA, RF, and anti-EPO in the sera.	1. Positive results in 82% EGPA patients with sensitivity and specificity of 82 and 44%, respectively, pre-dominated in males and associated with skin involvement. 2. Most positive patients had a negative IFT for ANCA. 3. There was no association between the presence and levels of anti-alpha-enolase antibodies and EGPA activity. 4. None of the EGPA patients and controls was positive for anti-EPO.	Alpha-enolase may be a target of autoimmunity in EGPA and usually shows negative ANCA IFT results.	Laskari et al. (144)
A panel of 22 proteins	Different types of vasculitis, including 37 patients with EGPA (most patients were on treatment).	A panel of 22 serum proteins was evaluated.	In EGPA G-CSF, GM-CSF, IL-6, IL-15 and sIL-2Rα showed significant increases during active disease, as did BCA-1/CXCL13 but only after adjustment for treatment.	1. G-CSF, GM-CSF, IL-6, IL-15, sIL-2Rα, and BCA-1/CXCL13 have been identified as a novel biomarkers of disease activity in GCA and EGPA. 2. Differences of biomarker levels between diseases independent of disease activity, were more apparent than differences related to disease activity.	Rodriguez-Pla et al. (145)
Anti-PTX 3 antibodies	EGPA: 38 GPA: 51 MPA: 12 SLE: 130 CTD: 97 Healthy controls: 97	Evaluation of anti-pentraxin 3 antibodies in the sera of all groups.	1. Anti-PTX3 antibodies were detected in 29.7% AAV patients, significantly more common in EGPA (44.7% vs. 25 and 19%). 2. The presence of anti-PTX3 was associated with a lower prevalence of systemic, ENT, and renal manifestations. 3. Among ANCA negative patients, 35.7% displayed positive anti-PTX3 antibodies. 4. The prevalence of anti-PTX3 antibodies was significantly higher in AAV patients than in healthy controls and other CDT patients, but lower than in SLE.	Anti-PTX3 antibodies appear a promising novel biomarker of AAV, especially of EGPA.	Padoan et al. (146)
Eicosanoid profile	EGPA: 23 Asthma: 30 HES: 12 Healthy controls: 54	Assessment of eicosanoid profile (18) in EBC of all groups; furthermore, in 21 of 23 EGPA patients and in 9 asthmatics eicosanoids were evaluated using BALF.	1. Markedly elevated levels of 12-HETE was found in EBC from EGPA compared to other groups 2. BALF was characterized by a significant elevation of 12-HETE and its metabolite 12-tetranor HETE in EGPA as compared with asthma, and correlated with disease activity.	12-HETE concentration in both EBC and BALF distinguish EGPA from asthma and HES.	Szczeklik W. et al. (147)
IL-33, sST2, TSLP, ILC2, blood eosinophil count	EGPA: 86 CEP: 25 Asthma: 11	Evaluation of serum levels of IL-33, sST2 and TSLP, and peripheral blood ILC2 count.	1. Blood eosinophil count or ILC2 and, sST2 or TSLP, and IL-33 were significantly higher in active EGPA than in inactive, at relapse, or in other diseases. 2. EGPA activity correlated with IL-33 and ILC2, but eosinophil count correlated with ILC2 TSLP (but not IL-33).	Increased ILC2 and IL-33 are associated with EGPA activity. Increases in IL-33 may indicate the presence of active vasculitis rather than peripheral or tissue eosinophilia.	Tsurikisawa et al. (45)
A panel of 160 protein	EGPA: 28 (13 active, 15 inactive).	The expression of 160 proteins was compared in sera from active and inactive EGPA	1. 12 out of 19 candidate markers were positively correlated with blood eosinophil count (FGF-7, SCF, GDNF, ß-NGF, IGFBP-4, Axl, PIGF, insulin, NT-4, ErbB3, OPN, BMP-4), while two, CD14 and MCP-3, were negatively correlated 2. The higher expression of Axl, OPN, HCC-4, GDNF, MCP-3 was found in active EGPA	The serum protein profiles were significantly different between active and inactive EGPA, however, Axl, OPN, HCC-4, GDNF and MCP-3 were consistently higher in active disease, with Axl having the largest AUC, indicating that it could be a candidate for a new biomarker of active EGPA.	Ma et al. (148)
Blood eosinophil count, ECP, IL-5, IL-4, IgG4, IgE, ANCA, periostin, IL-8, GM-CSF	EGPA: 30 (active) Severe eosinophilic asthma: 49	Evaluation of blood eosinophil count, and sera levels of ECP, IL-5, IL-4, IgE, IgG4, ANCA, and sputum biomarkers (eosinophils, periostin, IL-8, GM-CSF) to differentiate severe asthmatic patients from the prodromal phase of EGPA.	1. Patients with asthma had higher levels of sputum eosinophils, however, EGPA patients had higher levels of blood eosinophils in the past. 2. The GM-CSF was the only biomarker significantly increased in EGPA compared with asthma	Sputum GM-CSF might be a good biomarker of systemic eosinophilic disease.	Latorre et al. (149)
SAA1, FGA, SAP, CETP	EGPA: 58 Asthma: 33 Healthy controls: 25	Data-independent acquisition (DIA) followed by parallel reaction monitoring (PRM) analysis were performed to screen biomarkers for early diagnosis of EGPA and to differentiate asthma diagnosis.	1. Four candidate biomarkers were identified. SAA1, FGA, and SAP were upregulated in EGPA (sensitivity 82.3%, specificity 100%), while CETP was downregulated in EGPA compared to asthma. 2. The combination of SAA1, FGA, and SAP had a sensitivity and specificity of 82.35 and 100%, respectively, as biomarkers for early diagnosis of EGPA. 3. The combination of SAA1, FGA, SAP, and CETP had a sensitivity and specificity of 78 and 100%, respectively, as biomarkers for differential diagnosis of asthma.	SAA1, FGA, SAP, and CETP can be potentially useful biomarkers for early diagnosis of EGPA and differential diagnosis of asthma.	Xiao et al. (150)
FeNO, blood eosinophil count/percentage, total IgE	EGPA: 44 Allergic asthma: 44	Assessment of FeNO, eosinophil blood count/percentage, and total IgE for early diagnosis of EGPA and to distinguish EGPA from allergic asthma	1. FeNO level, blood eosinophil count/percentage, and total IgE were significantly higher in EGPA than in allergic asthma 2. Unlike the allergic asthma, there was no correlation between FeNO level and blood eosinophil count/percentage in EGPA	Patients with allergic asthma and high blood eosinophil count should be alert to the possibility of having EGPA For patients with infiltration of eosinophils into the airway, a diagnosis should not be based on peripheral blood eosinophil count (blood eosinophil count cannot predict eosinophilic airway inflammation and pulmonary function for patients with EGPA) FeNO level and PFTs should be monitored for patients who present with symptoms in other body systems	Zhao et al. (151)

EGPA, eosinophilic granulomatosis with polyangiitis; GPA, granulomatosis with polyangiitis; MPA, microscopic polyangiitis; AAV, antineutrophil cytoplasmic antibodies vasculitis; BVAS, Birmingham Vasculitis Activity Score; BVAS/WG, Birmingham Vasculitis Activity Score/Wegener Granulomatosis; GCA, giant cell arteritis; HES, hypereosinophilic syndrome; SLE, systemic lupus erythematosus; SSc, scleroderma; CU, colitis ulcerosa; CTD, connective tissue disease; CEP, chronic idiopathic pneumonia; SNPs, single nucleotide polymorphism; IgE, immunoglobulin E; IgG4, immunoglobulin G4; ESR, erythrocyte sedimentation rate; CRP, C-reactive protein; ENT, ear, nose, throat; CS, corticosteroids; IS, immunosuppressant; BALF, bronchoalveolar lavage fluid; EBC, exhaled breath condensate; 12-HETE, 12-hydroxy-eicosatetraenoic acid; ANCA, antineutrophil cytoplasmic antibodies; ANA, anti-nuclear antibodies; RF, rheumatoid factor; ECP, eosinophil cationic protein; IL4,5,6,8,15,33, interleukin, 4,5,6,8,15,33; sIL-2Rα, soluble IL-2 receptor alpha; MDC, macrophage-derived chemokine; MIP, 1α and 1ß-macrophage inflammatory protein 1alpha and 1beta; TSLP, thymic stromal lymphopoietin; CCL17/TARC, thymus and activation-regulated chemokine; TNF, α-tumor necrosis factor alpha; MCP-3, monocyte chemotactic protein 3; FGF-7, fibroblast growth factor 7; GM-CSF, granulocyte-macrophage colony-stimulating factor; G-CSF, granulocyte-colony stimulating factor; anti-PTX, anti-pentraxin 3 antibodies; anti-EPO, anti-eosinophil peroxidase antibodies; IFT, immunofluorescence test; BCA-1/CXCL13, B-lymphocyte chemoattractant; BMP-4, bone morphogenetic protein 4; sST2, soluble suppression of tumorigenicity 2 protein 2; ILC2, innate lymphoid cells 2; SCF, stem cell factor; GDNF, glial cell line-derived neurotrophic factor; ß-NGF, beta nerve growth factor; IGFBP-4, insuline-like growth factor-binding protein 4; PIGF, phosphatidylinositol-glycan biosynthesis class F protein; CD14, cluster of differentiation 14; OPN, osteopontin; NT-4, neurotrophin 4; ErbB3, receptor tyrosine kinase 3; Axl, receptor tyrosine kinase; HCC-4, human beta chemokine; SAA1, serum amyloid A1; FGA, fibrinogen alpha chain; SAP, serum amyloid P; CETP, cholesteryl ester transfer protein; FeNO, fractional exhaled nitric oxide; PFTs, pulmonary function tests.

In EGPA, each organ may be affected; therefore, it is essential to conduct a thorough medical history interview and perform diagnostic tests assessing the functions and/or organ lesions. In addition, it is important to detect life-threatening organ involvement, as it requires rapid implementation of treatment (153). Generally, once EGPA is diagnosed, evaluating possible lung, heart, kidney, GI, and peripheral nerve involvement is recommended (153). Regarding the lungs and respiratory manifestations, a complete pulmonary diagnostic evaluation, comprising chest imaging at baseline and pulmonary function tests, should be performed (153). Every patient should have at least one chest radiograph, however, a CT scan is more sensitive and can provide a more precise assessment of lung lesions (153). Bronchoscopy with an evaluation of inflammatory cells in BALF can confirm pulmonary eosinophilia (defined as ≥25% eosinophils at differential cell count) (108). When DAH is present, BALF is bloodier and contains hemosiderin-laden macrophages (5).

**FIGURE 1:**
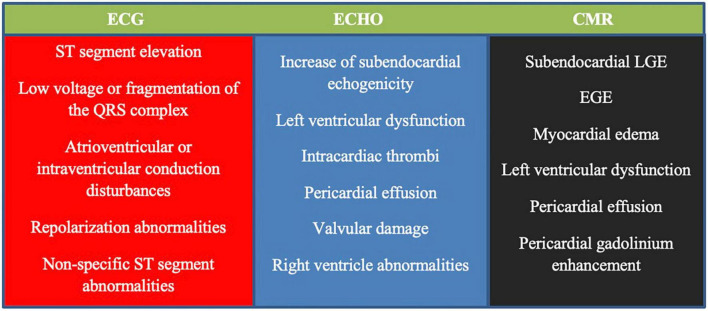
Signs of heart involvement in cardiological tests in patients with eosinophilic granulomatosis with polyangiitis (EGPA) [based on Bond et al. (155)]. ECG, electrocardiogram; ECHO, echocardiogram; CMR, cardiac magnetic resonance; LGE, late gadolinium enhancement; EGE, early gadolinium enhancement.

**FIGURE 2:**
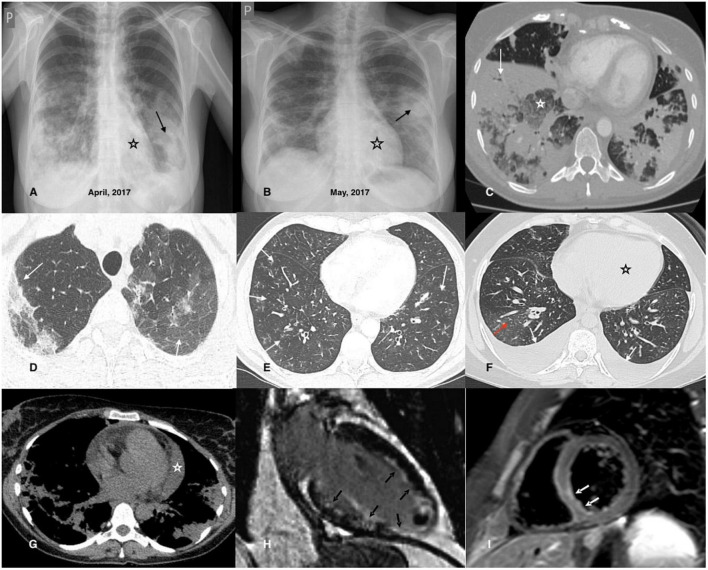
Chest imaging findings in patients with EGPA. **(A,B)** Chest X-rays of a 42-year-old female patient diagnosed with EGPA. They demonstrate migrating patchy infiltrates with peripheral dominance (black arrow), characteristic for eosinophilic infiltrates, and rapidly enlarging heart related to its acute injury in the course of EGPA. **(C)** Chest CT axial image (lung window) of a 40-year-old female EGPA patient showing pre-dominant massive bilateral ill-defined areas of airspace (white arrow) and ground-glass (white asterisk) opacities located in both lower lobes of the lungs. **(D)** Chest CT axial image (lung window) of a 37-year-old female patient presenting pre-dominant areas of ground-glass opacities of varying intensity in both upper lobes of the lung (black arrows); histological examination of the transbronchial biopsy specimen reveals features of eosinophilic pneumonia and eosinophilic vasculitis. **(E)** The image refers to a 46-year-old male patient admitted for worsening asthma and eosinophilia. Chest CT axial image (lung window) shows a pre-dominant airway pattern-bronchi wall thickening and small centrilobular nodules (white arrows). BALF examination indicated pulmonary eosinophilia (65% of eosinophils), and the patient complained of numbness of the feet, and for several days purpura-type skin lesions occurred; MPO-ANCA was detected in the sera. The patient was diagnosed with EGPA. **(F)** Chest CT axial image (lung window) of a 34-year-old female patient with EGPA and cardiac involvement showing pre-dominant features of cardiac insufficiency; the ground-glass opacities with interlobular septal thickening (red arrow) corresponding to interstitial edema; bilateral pleural effusion (white arrow) and enlarged heart is also present (black asterisk). **(G)** Chest CT axial image (mediastinal window) of a 38-year-old female patient diagnosed with EGPA and cardiac involvement. In addition to bilateral pulmonary infiltrates, an enlarged heart and pericardial effusion is visible (white asterisk). **(H)** CMR refers to a 32-year-old male patient with EGPA; late gadolinium enhancement (LGE) image in vertical long axis cross-section showing subendocardial enhancement pattern (typical for EGPA) of the anterior wall, subendocardial and transmural enhancement of the inferior wall, inferior papillary muscle and the left ventricle (LV) apex (black arrows); thrombus seen as an unenhanced mass in the apical part of the LV cavity. **(I)** CMR refers to a 26-year-old male patient diagnosed with EGPA with cardiac involvement; a T2-weighted turbo spin-echo (TSE) image with fat saturation in the short axis mid-cavity cross-section, presenting edema in the infero-lateral segment of the LV (white arrows).

Cardiac involvement, in particular, is associated with poor prognosis (114, 154); therefore, basic cardiological examinations are recommended in all patients (at diagnosis and in case of relapse), irrespective of clinical symptoms (118, 153, 155). These examinations include resting electrocardiography (ECG), echocardiography (ECHO), and serum concentrations of brain natriuretic peptide (BNP) and troponin (118, 153, 155). The 24-h ECG monitoring can help detect arrhythmias that cannot be captured on resting ECG and may be life-threatening, leading to sudden death. Recently, cardiac magnetic resonance (CMR) imaging has been considered the gold standard technique for evaluating cardiomyopathies (118, 121, 156). It is a safe and non-invasive tool for the assessment of cardiac involvement in AAVs (118, 121, 122, 156–158). Furthermore, it can help identify the individual stages of myocarditis (with better visibility of endocavitary thrombosis) and determine the activity of the disease (121, 156, 158, 159), however, its particular diagnostic importance is in asymptomatic patients, in whom this manifestation can be easily overlooked (118, 120–123, 155, 158, 160, 161). CMR is also a useful tool for monitoring treatment efficacy and fibrosis (121, 161). Late gadolinium enhancement (LGE) by CMR (mostly of subendocardial location) is characterized by high sensitivity and specificity for the detection of cardiac inflammation and fibrosis (121), and its persistence following treatment has become a marker of cardiac disease severity (112). However, CMR abnormalities are detected in a high proportion of patients in clinical remission and their clinical and prognostic significance remains unclear (123, 161). Although endomyocardial biopsy (EMB) is still considered the gold standard for the diagnosis of myocarditis, it is not routinely performed due to the risk of complications and organizational difficulties. This procedure may be considered in doubtful cases, especially, when the diagnosis of EGPA has not been established (155). Signs of heart involvement in cardiological tests in EGPA are demonstrated in Figure 1. Figure 2 presents chest imaging findings in patients with EGPA.

Renal involvement is the next poor prognostic factor; therefore, renal function tests and urinalysis should be performed in all cases at baseline and during follow-up (153). In asymptomatic patients, routine screening for GI and peripheral nerve involvement is not required, however, when symptoms are present, appropriate diagnostic procedures should be implemented (e.g., radiologic and/or endoscopic evaluation of the digestive tract in cases of gastrointestinal symptoms or electromyography and nerve conduction studies in cases suspected of nerve involvement). Other evaluations should be guided by clinical symptoms and physical examination (153).

In the presence of demonstrable lesions, biopsy procedures should be considered when feasible, and the patient’s condition allows it, however, histological examination is not strictly necessary (153). Although pathomorphological lesions are well-defined (necrotizing vasculitis, extravascular granulomas, and eosinophil infiltration of arterial walls and adherent tissue), it is extremely rare to find all of them simultaneously (<20% of patients) (135). The most commonly biopsied organs are the skin, nerves, and muscles. Although EGPA is considered a multi-organ disease, it is well known that limited forms may also occur. When a single extrapulmonary manifestation attributable to systemic disease is present, the disease may be called “formes frustes” of EGPA (108). In such situations, diagnosis is only possible by organ biopsy (162).

While EGPA share features with eosinophilic inflammation and vasculitis, the primary differential diagnoses include other eosinophil-related disorders and vasculitides. First, other common causes of secondary eosinophilia should be excluded from the study. Eosinophilia can be reactive to drugs, and severe reactions may result in organ manifestations mimicking EGPA (e.g., drug rash with eosinophilia and systemic symptoms, DRESS syndrome) (16). A careful history of medication use is crucial to emphasize the association between drug use and symptom onset. Second, helminthic infections need to be ruled out. Serology of Toxocara and *Strongyloides stercoralis* is especially recommended (153). Both are associated with high eosinophilia and can be clinically inapparent (163, 164). Other parasite investigations depend on the patient’s country of origin and travel history, however, stool culture, although it has low sensitivity, should also be performed (16). Next, screening for HIV should be performed, even though eosinophilia in this infection is usually mild (153). Lymphocytic variant reactive hypereosinophilia should also be considered, especially when skin manifestations dominate, with accompanying hypergammaglobulinemia. In such cases, lymphocyte immunophenotyping and T-cell receptor rearrangement analysis are indicated (153, 165).

Eosinophilic granulomatosis with polyangiitis often manifests as respiratory symptoms and lung infiltrates; therefore, it should be differentiated from eosinophilic lung disorders. ABPA and idiopathic EP share many features with EGPA, including eosinophilia, cough, dyspnea, and lung infiltrates. Moreover, a large proportion of patients with these diseases have asthma, which is a cardinal feature of EGPA (166, 167). ABPA is characterized by elevated serum *Aspergillus fumigatus*-specific IgE and IgG concentrations (149, 162) and often isolated fungal cultures in sputum or BALF (153, 166). However, distinguishing idiopathic EP from the second stage of EGPA remains challenging. The lack of organ symptoms and ANCA may help differentiate between the two (5), however, patients with idiopathic EP should be monitored for extrapulmonary symptoms because they may develop EGPA in the future.

Hypereosinophilic syndromes are the next most important consideration in the differential diagnosis of EGPA, given the overlapping clinical, radiologic, and histologic features, and biomarker profile (105, 168). Depending on the pathogenesis, three main types of HESs are distinguished: reactive (rHES), neoplastic (nHES), and idiopathic (iHES). In rHES, eosinophils are non-clonal and are thought to be driven by Th2 cytokines, mainly IL-5. This group includes patients with classified conditions associated with secondary eosinophilia, including EGPA (11, 165, 169) (however, eosinophilia in EGPA is not entirely secondary, as it has a partially genetic background related to the IRF1/IL5 gene variant) (56). In nHES, eosinophils are clonal and derived from eosinophil progenitors containing genetic alterations in oncogenic tyrosine kinase receptors, such as platelet-derived growth factor receptor A (PDGFRA) and B (PDGFRAB) and fibroblast growth factor receptor 1 (PGFR1) (105, 165, 169). This group also encompasses other myeloid neoplastic diseases with associated eosinophilia (with or without genetic abnormalities), as well as chronic eosinophilic leukemia. In turn, iHES is the largest type of HES (comprising about 50% of cases) and is a diagnosis of exclusion once reactive and neoplastic causes have been excluded (105, 165).

Although organ damage may be similar, some symptoms, such as hepatomegaly or splenomegaly, can be suggestive of clonal eosinophilia and nHES. In addition, a proportion of patients have abnormal peripheral blood counts, such as anemia (53%) or thrombocytopenia (31%), and patients with nHES usually do not respond to treatment with systemic CS (169). Screening for serum vitamin B12 and tryptase levels is sensitive to nHES and is recommended for all patients diagnosed with eosinophilia (153). In cases of suspected nHES, fusion gene testing is indicated. However, although only to be positive in nHES, a case of PDGFRA-positive EGPA has been described (42); therefore, some authors believe that testing for PDGFRA mutation should be performed routinely in all cases with hypereosinophilia, regardless of clinical manifestation, suspected EGPA, or ANCA-status (170).

Idiopathic HES is the most difficult to distinguish from EGPA, especially in ANCA-negative cases without vasculitic symptoms (165, 169, 171, 172). Both clinical and radiological symptoms are similar, but HES is usually not considered to have asthma or nasal polyps. However, this is not a distinguishing feature. A case series of iHES with the first presenting asthma-like symptoms has been recently described (173). In addition, it has been reported that approximately 10% of patients with HES have rhinitis (169). Histological examination also showed no differentiation. HES is typically characterized by tissue infiltration by eosinophils, which is also often found in cases of EGPA (105). Other findings, such as vasculitis and granulomas, are not typical for HES but are considered hallmark features of EGPA (2). Recently, among patients with a diagnosis of HES lacking asthma, a group characterized by necrotizing eosinophilic vasculitis confirmed by biopsy has been distinguished (174). The distinction of EGPA from this entity is challenging, especially because it cannot be excluded that both may be a part of a common spectrum.

There is a need for further research on suitable features for distinguishing EGPA from HES. Finally, a comparative study of 166 patients with blood eosinophilia (>1.000 cells/μL) and systemic manifestations demonstrated that CRP level was a sound diagnostic biomarker that could accurately differentiate between HES and EGPA, with low levels (<36 mg/L) suggestive of HES (175). Other authors have proposed a HES-suggesting laboratory index (HSLI) based on white and eosinophil blood count, with values ≥4.25 exhibiting a significantly high relative risk for HES (176). Recently, a scoring system (E-CASE) for differentiating EGPA from other types of eosinophilic disorders, including HES, has been proposed. It was based on the clustering analysis of 19 parameters of 58 patients with eosinophil-related diseases at a tertiary hospital and was extensively validated in 40 patients at another tertiary institution. This system includes clinical (peripheral nerve disorder, asthma, lung, and skin involvement), laboratory (RF positivity, MPO-ANCA positivity, IgE, and CRP elevation), and histological features (vasculitis detected by pathological examination), which have been awarded a point weight. A score ≥12 was considered positive for EGPA (177).

The next diseases that should be differentiated include other forms of vasculitis, especially AAVs. GPA and MPA share several clinical and histological features with EGPA, however, there is usually a lack of asthma and eosinophilia. Nevertheless, eosinophilia may be present in GPA, although it is usually modest, and there are some clinical features distinguishing it from EGPA (Table 4) (178–180). EGPA may also need to be differentiated from polyarteritis nodosa (PAN), a rare form of necrotizing vasculitis that preferentially targets medium-sized arteries. Hypereosinophilia may occasionally be observed in PAN, and similar to EGPA, skin and peripheral nerves are the most frequently affected tissues. However, PAN is not associated with glomerulonephritis and small-vessel involvement, and ANCA is typically negative. In addition, it may be triggered by viral infections, particularly the hepatitis B virus; thus, patients may have positive viral serology and histological granulomas are usually absent (181).

**TABLE 4:** Differentiating EGPA from other AAVs [data based on the Samson et al. (54), Comarmond et al. (55), Tsurikisawa et al. (83), Fijolek et al. (87), Saku et al. (101), Puechal (178), Greco et al. (179), Nguyen et al. (180), Liu et al. (185), and Papo et al. (186)].

		EGPA	GPA	MPA
Serological features	Peripheral eosinophilia	**++++**	+	–
	ANCA	MPO 30–40% PR3 2% ANCA (–) >60%	PR3 80–95% MPO 5–20% ANCA (–) 0–20%	MPO 70–80% PR3 30% ANCA (–) 0–20%
Clinical features	ENT	70–90% allergic rhinosinusitis, nasal polyposis, usually without destruction	80–93% often destructive, with ulcers, crusting, septal perforation, hearing loss	_
	Lungs/airways	38–77% not-fixed pulmonary infiltrates, small nodules without cavitations asthma: 90–100%	53–83% nodules with cavitation, ground-glass opacities, consolidations, subglottic stenosis	25–55% pulmonary fibrosis DAH
	Heart	11–76% (mortality) ANCA positive 12-18.5% ANCA negative 30-38.7%	4–40%	10–21%
	Digestive tract	19.8–78% ANCA positive 22–24% ANCA negative 23–34.4%	11–24%	30–58%
	Skin	23–68% ANCA positive 36–45.4% ANCA negative 36.3–53.8%	33–45%	30–60%
	Nerve involvement	42–74% ANCA positive 64–66.7% ANCA negative 43–47.5%	20–50%	37–72%
	Kidneys	16–35% ANCA positive 27–80% ANCA negative 16–35.5%	50–80%	80–100% (severe)
	Eyes	<5%	28–50%	<5%
Histological features	Granulomas	++++ dominated by eosinophils	++++ dominated by neutrophils	_

AAVs, antineutrophil cytoplasmic antibody-associated vasculitides; EGPA, eosinophilic granulomatosis with polyangiitis; GPA, granulomatosis with polyangiitis; MPA, microscopic polyangiitis; ANCA, antineutrophil cytoplasmic antibody; MPO, myeloperoxidase; PR3, proteinase 3; ENT, ear, nose, throat; DAH, diffuse alveolar hemorrhage.

Finally, because IgG4 may be elevated in a significant proportion of patients with EGPA (47, 48, 50), IgG4-RD has become an important differential diagnosis to consider. IgG4-RD may share some clinical features with EGPA, such as asthma, rhinitis, or peripheral eosinophilia (174, 182). Histopathologic examination is essential for diagnosis, which typically demonstrates lymphoplasmacytic infiltrate, storiform fibrosis, and obliterative phlebitis without vasculitis or granulomas (183).

### 5.3. Disease phenotypes

**FIGURE 3:**
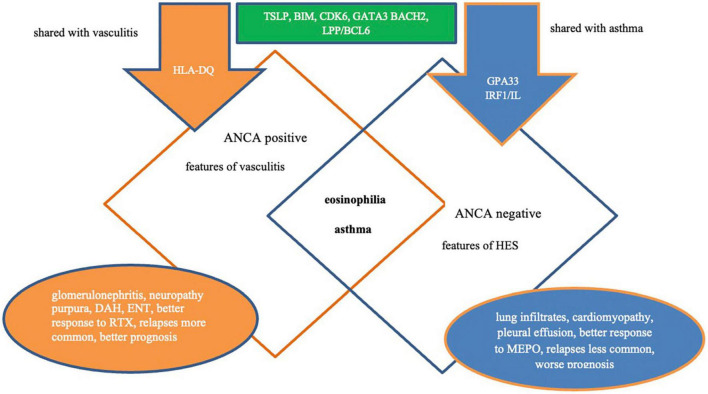
Phenotypes of EGPA. ANCA, antineutrophil cytoplasmic antibodies; DAH, diffuse alveolar hemorrhage; ENT, ear, nose, throat; MEPO, mepolizumab; RTX, rituximab; AAV, antineutrophil cytoplasmic antibody vasculitis; HES, hipereosinophilic syndrome.

Although ANCA is detected in only 30–40% of patients, two main phenotypes of EGPA have been identified according to ANCA status, differing in clinical features, treatment response, and prognosis (Figure 3). First, the “vasculitic” phenotype (associated with ANCA-positivity and vasculitis symptoms), and second – the “tissular” phenotype (associated with ANCA-negativity and organ damage related to eosinophilic inflammation) (9, 10, 55, 100, 101, 184), both confirmed using GWAS, which found the distinct genetic background for each of them (56). However, these phenotypes rarely occur separately and tend to overlap in the same patient (128, 159). Generally, patients with positive MPO-ANCA have a more active disease with higher CRP levels, higher ratios of fever and myalgia (185), and significantly more common rhinosinusitis (184) than those with negative MPO-ANCA. However, the pre-valence of asthma does not appear to be dependent on ANCA status (184), although in some studies asthma was more common in ANCA-negative patients (185).

The next specific subgroup of EGPA patients was those with PR3-ANCA positivity. ANCA directed against PR3 is much less common in EGPA patients. In a recent large retrospective study of 734 patients with EGPA, PR3-ANCA was detected in 2% of cases and has been associated with a distinct clinical profile with features reminiscent of GPA (186). Compared to those with MPO-ANCA and ANCA-negative, patients with PR3-ANCA less frequently had asthma and peripheral neuropathy, while more frequently had skin symptoms, pulmonary nodules, and a lower median eosinophil count. Interestingly, myocarditis in this group was observed as frequently as in ANCA-negative patients and more frequently than in MPO-ANCA patients. In turn, long-term outcomes, such as relapse-free survival and overall survival in PR3-ANCA-positive EGPA patients were similar to those in patients with GPA PR3-ANCA (186).

## 6. Therapeutic management

The treatment strategy for EGPA depends on the severity of the disease (Table 5) and consists of induction and maintenance phases. The first phase aims to achieve remission of the disease, whereas the second phase prevents relapses. Other important objectives of treatment include limiting side effects and sequelae, improving the quality of life, and enabling the rapid return of the patient to normal activities (187). Prospective clinical trials specifically dedicated to EGPA are limited (188). Thus, treatment recommendations have been mostly derived from the results of trials involving other AAVs, rather than EGPA itself, and/or are based on expert opinion (153, 187).

**TABLE 5:** Eosinophilic granulomatosis with polyangiitis severity criteria according to the FVSG and ACR.

FVSG		ACR	
FFS 1996	FFS 2009	Severe disease	Non-severe disease
1. Proteinuria > 1 g/d 2. GI bleeding, perforation, infarction, and/or pancreatitis 3. Renal insufficiency (Cr > 158 mg/dL) 4. CNS involvement 5. Cardiomyopathy The 5-years mortality rates: FS = 0 11.9% FFS = 125.9% FFS ≥ 245.95% study population: EGPA 82 MPA 52 PAN 260	1. Age > 65 years 2. Cardiac insufficiency 3. Renal insufficiency (stabilized peak Cr ≥ 150 μmol/L) 4. GI 5. Absence of ENT The 5-years mortality rates: FFS = 09.0% FFS = 121% FFS ≥ 240% study population: EGPA 230 MPA 218 PAN 349 GPA 311	Vasculitis with life- or organ-threatening manifestations, e.g., DAH, glomerulonephritis, central nervous system vasculitis, mononeuritis multiplex, cardiac involvement, mesenteric ischemia, limb/digit ischemia	Vasculitis without life- or organ-threatening manifestations, e.g., rhinosinusitis, asthma, mild systemic symptoms, uncomplicated cutaneous disease, mild inflammatory arthritis
FFS = 0 non-severe disease FFS ≥ 1 severe disease DAH, eye involvement, fulminant mononeuritis multiplex are also categorized as severe disease, although not listed in FFS			

FFS, Five-Factor Score; PAN, polyarteritis nodosa; EGPA, eosinophilic granulomatosis with polyangiitis; MPA, microscopic polyangiitis; GPA, granulomatosis with polyangiitis; GI, gastrointestinal involvement; Cr, creatinine; CNS, central nervous system; ENT, ear, nose, throat; DAH, diffuse alveolar hemorrhage; ACR, American College of Rheumatology; FVSG, French Vasculitis Study Group.

### 6.1. Conventional agents

Induction therapy should be adapted according to disease severity (153, 187). Systemic CS is the cornerstone drug in EGPA, and treatment with CS alone is justified in patients with an Five-Factor Score (FFS) of 0 (153, 187). The initial recommended dose is 1 mg/kg/day of prednisolone equivalent with a maximum dose of 60 mg/kg/day for 2–3 weeks, followed by gradual reduction to the minimal effective dose or, if possible, until withdrawal (153). In severe cases with life-threatening manifestations, methylprednisolone pulses can be applied (at a dose of 7.5–15 mg/kg/day for 3 days, followed by oral CS) (153); however, there are no data to support favoring either intravenous pulse or high-dose oral CS for active severe EGPA (189). The French Vasculitis Study Group (FVSG) proposes a tapering-off schedule of CS between 12 and 18 months, of which the reference doses are around 20 mg/day, 10 mg/day, and 5 mg/day at 3 months, 6 months, and 1 year, respectively, of prednisolone equivalent (187), however, the threshold to which CS can be reduced without compromising asthma and/or ENT symptoms is unknown and varies from patient to patient. Optimization of local therapies may help reduce the risk of flares during oral CS tapering (e.g., increasing the dose of inhaled CS or nasal CS implementation) (190), while new biological therapies can make a significant contribution to lowering the dosage of maintenance CS therapy.

In cases with at least one poor prognostic factor (FFS ≥ 1), combined treatment with CS and IS is recommended (153, 187). No randomized controlled trial results are available to support this recommendation, however, the benefit of adding intravenous cyclophosphamide (CYC) to CS to achieve remission has been demonstrated (191). The preferred immunosuppressant is intravenously administered CYC at a dose adjusted for age and renal function (Table 6) (192). The FVSG guidelines recommend a dose of 0.6 g/m^2^/per infusion on days 1, 15, and 30, followed by a dose of 0.7 mg/m^2^/per infusion every 3 weeks, with a maximum of 1.2 g per infusion (153, 187). In cases with impaired kidney function (<65 years of age), treatment should be started with a lower dose of 0.5 g/m^2^/per infusion, while in elderly patients, a rigid dose of 0.5 g/per infusions is recommended (regardless of kidney status) (153, 187). CYC infusion should be combined with antiemetic therapy and good hydration, with 2-mercaptoethanesulfonate sodium (MESNA) prophylaxis to limit bladder toxicity (190). In addition, CYC can also be administered orally at a dose of 2 mg/kg/day (without exceeding 200 mg/day) for 3–6 months, however, intravenous treatment is preferred due to better compliance and lower cumulative drug dose (190). During the IS treatment, prophylaxis of *Pneumocystis jirovecii* is indicated (co-trimoxazole 400 mg/day or 980 mg thrice weekly), and screening for drug-induced neutropenia is necessary. The patient should be informed about the need for contraception and the possibility of egg/sperm freezing (153, 187, 190). If remission is achieved, maintenance treatment should be started 2–3 weeks following the last CYC pulse or a few days after oral CYC. The preferable drug is azathioprine (AZA), at a dose of 2–3 mg/kg/day, followed by methotrexate (MTX) at a dose of 0.3 mg/kg/week, for 18–24 months (153, 187, 190).

**TABLE 6:** Dosing of CYC pulses depending on age and renal function (EULAR).

Age (yrs)	Creatinine (umol/L)	
	<300	300–500
<60	15 mg/kg/pulse	12.5 mg/kg/pulse
60–70	12.5 mg/kg/pulse	10 mg/kg/pulse
>70	10 mg/kg/pulse	7.5 mg/kg/pulse

CYC, cyclophosphamide; EULAR, European League against rheumatism.

In cases of severe DAH, eye involvement, or fulminant mononeuritis multiplex, IS induction treatment should also be considered, although it is not listed in the FFS (153).

As previously mentioned, in patients without poor prognostic factors (FFS = 0), IS treatment in the induction phase is not indicated. This is supported by the results of the CHUSPAN 2 study, which demonstrated that adding AZA to CS in these patients did not improve remission rates, lower relapse risk, spare steroids, or diminish EGPA asthma or ENT exacerbation rates (193). However, treatment with IS as a second-line therapy can be considered in the group in two clinical situations: first, as a CS-sparing treatment in cases of CS dependence of >7.5–10 mg/day; second, in cases of CS intolerance. The preferred drugs are AZA and MTX, according to the scheme mentioned above (153, 187).

The recently published ACR guidelines differ from those of the FVSG (Table 7). According to these guidelines, the addition of an adjunctive IS is recommended in all patients with EGPA as the first-line therapy, regardless of the disease severity (not based on FFS), to reduce CS toxicity, however, no study results support this strategy (194).

**TABLE 7:** Key recommendations for the treatment of EGPA according to the FVSG and ACR taking into account biologics.

	Severe disease	Non-severe disease	Severe relapse	Non-severe relapse
FVSG	CS + CYC 3–6 months (induction phase) AZA or MTX ≥18 months (maintenance phase)	CS alone to minimal dosage or withdrawal	RTX can be considered, especially after CYC failure (in induction phase) RTX can be considered after AZA or MTX failure (in maintenance phase)	CS + MEPO (first choice) CS + AZA or MTX
ACR	CS + CYC or RTX if remission on CYC, switch to MTX or AZA or MMF	CS + MEPO (first choice) CS + MTX/AZA/MMF CS + RTX CS alone in selected patients	CS + RTX	CS + MEPO

EGPA, eosinophilic granulomatosis with polyangiitis; FVSG, French Vasculitis Study Group; ACR, American College of Rheumatology; CS, corticosteroids; CYC, cyclophosphamide; AZA, azathioprine; MTX, methotrexate; MEPO, mepolizumab; RTX, rituximab; MMF, mycophenolate mofetil.

### 6.2. Biological agents

In recent years, new treatment options for EGPA have emerged. The therapeutic array has expanded with the introduction of new biological drugs, which have been intensively studied (Table 8). Depending on the mechanism of action, these drugs can be divided into two groups: first, directed against eosinophilic inflammation; second, directed against the autoimmune component of EGPA and vasculitis. However, so far, no single agent allows complete control of EGPA, and the choice should be dictated by the clinical features (15).

**TABLE 8:** Completed and ongoing clinical trials with the use of biologics in EGPA.

Name	Start date	Aim	Cohort/intervention	Primary outcome	Results	Date of completion
MATOCSS NCT 00527566 open-label phase 1/2 *N* = 7	2007	Evaluation the safety and efficacy of MEPO as a steroid sparing treatment in patients with Churg-Strauss syndrome receiving stable steroid dose (at least 10 mg daily of prednisone or equivalent)	MEPO 750 mg iv every 4 weeks	Treatment-related side effects The lowest prednisone dose achieved at the end of the treatment phase	MEPO was well tolerated, and there were no severe AE There was a decrease in mean CS dose from 12.9 to 4.6 mg/day after 12 weeks of treatment	2009
MEPOCHUSS NCT 00716651 prospective open-label phase 2 *N* = 10	2008	Evaluation the efficacy and safety of MEPO for patients with refractory or relapsing Churg-Strauss syndrome	MEPO 750 mg iv every 4 weeks	Percentage of patients that attain remission (defined as BVAS = 0 and CS < 7.5 mg/day) at 32 week	Eight patients reached the remission at 32 week The daily CS dose was reduced in all patients No relapse occurred with MEPO therapy	2010
MIRRA NCT 02020889 prospective randomized double-blind phase 3 *N* = 136	2014	Investigating the efficacy and safety of MEPO in patients with EGPA receiving standard-of-care therapy.	MEPO 300 mg sc every 4 weeks vs. placebo	Number of patients in each group of the accrued duration of remission (defined as the number of weeks where BVAS = 0 and CS ≤ 4 mg/day over 52 weeks). The number of patients in remission at 36 and 48 weeks.	Accrued weeks of remission were significantly more in MEPO treated group than in the placebo group (28% vs. 3%). A higher percentage of MEPO-treated patients were in remission at both 36 and 48 weeks (32 vs. 3%). The annualized relapse rate was significantly lower in MEPO treated group than in the placebo group (1.14 vs. 2.27). 44% of MEPO-treated patients had a CS-sparing effect (vs. 7% in placebo). Remission did not occur in 47% of MEPO-treated patients.	2016 This study led to approval of MEPO for the treatment of EGPA
RITE NCT 02947945 prospective open-label phase 2 *N* = 10	2017	Evaluation of the efficacy and safety of reslizumab in the treatment of EGPA	All subjects received reslizumab at a dose of 3 mg/kg iv every 4 weeks for 28 weeks (in addition to standard of care therapy).	Safety of reslizumab in patients with EGPA.	Reslizumab was well tolerated and resulted in a significant reduction in daily oral CS. Of the 10 subjects, 3 experienced an EGPA exacerbation; one had a severe AE.	2018
BITE NCT 03010436 prospective open-label phase 2 *N* = 10	2017	Evaluation the efficacy and safety of benralizumab in the treatment of EGPA.	All patients received benralizumab at a dose of 30 mg sc every 4 weeks for 12 weeks, and then every 8 weeks for 16 weeks (in addition to standard-of-care therapy).	Safety and tolerability of benralizumab in patients with EGPA.	Benralizumab was well tolerated and resulted in reduction of median oral CS dose from 15 mg at the start to 2 mg at the end of treatment Mean annualized exacerbation rate was lowest during treatment compared with the pre- and post-treatment phases (1.5 vs. 4.6).	2019
NCT05030155 prospective randomized double-blind phase 3 *N* = 100	2022	Comparison of MEPO-based regimen to conventional treatment for remission induction in EGPA.	Newly diagnosed or relapsed active EGPA (BVAS ≥ 3), FFS = 0 lub ≥ 1, not exceeding the first 21 days of CS therapy; arms: MEPO 300 mg sc every 4 weeks vs. placebo (FFS = 0) MEPO 300 mg iv every 4 weeks vs. CYC iv followed by AZA p.o. (FFS ≥ 1), CYC iv followed by AZA p.o. vs. placebo group.	Percentage of patients who achieved a CS dose of ≤ 4 mg per day at day 168 without experiencing a relapse.	Ongoing	2025
Long-term Access Program (LAP) of mepolizumab for subjects who participated in study MEA115921 NCT 03298061 open-label phase 3 *N* = 104	2015	Assessment a long-term efficacy of MEPO in the treatment of EGPA in patients receiving standard-of-care therapy.	Patients who require a dose of CS of 5 mg per day will receive MEPO at a dose of 300 mg sc every 4 weeks.	Number of patients with CS use (up to 3 years); number of AEs.	Ongoing	2023
MANDARA NCT 04157348 prospective randomized double-blind phase 3 *N* = 140	2019	Assessment the efficacy and safety of benralizumab compared to MEPO in the treatment of EGPA in patients receiving standard-of-care therapy.	Benralizumab 30 mg every 4 weeks sc vs. placebo and MEPO 300 mg sc every 4 weeks vs. placebo.	Proportion of patients who are in remission at both 36 and 48 weeks (BVAS = 0 and CS ≤ 4 mg per day, or BVAS = 0 and CS ≤ 7.5 mg per day).	Ongoing	2024
OCEAN NCT05263934 prospective randomized double-blind phase 3 *N* = 160	2022	Assessment the efficacy and safety of depemokimab compared with MEPO in relapsing or refractory EGPA in patients receiving standard-of-care therapy.	Depemokimab 200 mg sc every 26 weeks and placebo and MEPO 300 mg sc every 4 weeks.	Number of patients with remission at both 36 and 52 weeks (BVAS = 0 and CS ≤ 4 mg per day)	Ongoing	2025
REOVAS NCT 02807103 prospective randomized double-blind Phase 3 *N* = 105	2016	Comparison of RTX-based regimen to conventional treatment for remission induction in newly diagnosed or relapsing EGPA.	Arms: in FFS = 0: CS + RTX 1 g in D1 and 15 or CS + placebo; in FFS ≥ 1: CS + RTX or CS + CYC iv at a dose of 600 mg/m2 at 115, and 29 days, then 500 mg-fixed dose every 3 weeks (together 9 pulse).	The percentage of patients who obtained remission at day 180 (BVAS = 0 and CS ≤ 7.5 mg per day)	The remission rates in patients treated with RTX were comparable to those treated conventionally (63.5 vs. 60.4%). The mean duration of remission was comparable between two groups (10.37 vs. 11.68 weeks). VDI tended to be better in RTX-treated group.	2020
MAINRITSEG NCT 03164473 prospective randomized double-blind phase 4 *N* = 98	2018	Comparison of the efficacy and safety of RTX to AZA for maintenance remission in newly diagnosed or relapsing EGPA.	Arms: RTX at a dose of 500 mg iv every 6 months (4 infusions) and placebo vs. AZA 2 mg/kg per day for 24 months and placebo.	Duration of remission (BVAS = 0 and CS ≤ 7.5 mg per day) in weeks (time frame: 28 months).	Ongoing	2025

CS, corticosteroids; EGPA, eosinophilic granulomatosis with polyangiitis; FFS, Five-Factor Score; AZA, azathioprine; CYC, cyclophosphamide; MEPO, mepolizumab; RTX, rituximab; BVAS, Birmingham Vasculitis Activity Score; VDI, Vasculitis Damage Index; ENT, ear, nose, throat; AEs, adverse events.

#### 6.2.1. Anti-eosinophil-driving cytokines agents

In the pathogenesis of EGPA, eosinophils play a key role; therefore, agents inhibiting these cells may be effective. IL-5 is the main cytokine that drives eosinophil maturation and proliferation (21). The first clinical evidence of the successful use of mepolizumab—a monoclonal antibody that prevents the binding of IL-5 to its receptor—was described in 2010 in two distinct studies of patients with EGPA treated with mepolizumab infusions at a dose of 750 mg monthly (195, 196). The breakthrough was the randomized MIRRA trial investigating the safety and efficacy of mepolizumab at a dose of 300 mg s.c. monthly as an add-on therapy in 136 EGPA patients with relapsing or refractory disease. In that study, compared to placebo, patients treated with mepolizumab had significantly more accrued weeks of remission (28 vs. 3%, OR 5.91 for ≥24 weeks of accrued remission; *p* > 0.001) and a higher rate of remission at both weeks 36 and 48 (32 vs. 3%, OR 16.74; *p* < 0.001). Relapses at 52 weeks were less frequent (56 vs. 82%; *p* < 0.001), and the average dose of oral CS was lower in the mepolizumab group between weeks 48 and 52 (44 vs. 7%; *p* < 0.001). Importantly, there were no differences in drug safety between the two arms (23). This trial led to the approval of mepolizumab by the Food and Drug Administration (FDA) in 2017 as the first biologic drug for the treatment of EGPA.

Despite these promising results, the MIRRA trial has several limitations that need to be outlined. First, about half of the patients treated with mepolizumab did not achieve protocol-defined remission as the Birmingham Vasculitis Activity Score (BVAS) = 0 and less than 4 mg of daily prednisone (however, a *post-hoc* analysis, in which a comprehensive definition of clinical benefit was applied, revealed that 78–87% of patients experienced benefit with mepolizumab) (197); second, the diagnostic criteria of EGPA were very loose and active asthma was considered a feature of EGPA relapse; third, none of the patients received mepolizumab as first-line therapy (all were treated with oral CS with or without IS); finally, only 10% of the patients included in the study were ANCA-positive; therefore, the ability of mepolizumab to limit vasculitis could not be reliably assessed.

Mepolizumab is now considered a potential treatment for non-severe relapsing and/or refractory EGPA, with limited data available on its impact on vasculitic manifestations (198). It has been demonstrated that its effectiveness is not affected by the baseline treatment of EGPA, duration of disease, or refractory status of the disease (199). The expert panel of the FVSG recommends mepolizumab to treat EGPA patients whose asthma is CS-dependent (>7.5 mg/day) and/or ENT manifestation, starting at a dose of 100 mg monthly, which has been approved for the treatment of severe eosinophilic asthma and is three times lower than that approved for the treatment of EGPA (200). The use of mepolizumab in Europe at this dosage is currently off-label (198), but many real-life studies have shown positive results with low-dose mepolizumab in patients with EGPA (201–203). More recently, a retrospective collaborative study of 203 patients demonstrated that 100 mg can be an effective and safe dosage in EGPA and that its efficacy is comparable to that of 300 mg in remission rates, CS sparing effect, and rates of asthma/ENT exacerbations. In that study, improvement was observed in 10% of patients after dose escalation, suggesting that low-dose mepolizumab could be used as a first-line therapy with the possibility of an increase to 300 mg monthly in cases with an unsuitable response (204). Interestingly, out of 10 patients with CI treated with mepolizumab (at a dosage of either 100 or 300 mg/4 weeks), 9 achieved complete remission, suggesting that the inhibition of IL-5 signaling might be an effective novel treatment strategy for eosinophilic cardiac disease (204).

To date, no available data are evaluating the value of mepolizumab in the remission induction phase in patients with EGPA, however, some small retrospective studies have demonstrated that the use of mepolizumab as remission induction for severe EGPA might be safe and effective for controlling disease activity and reducing CS doses (205). Two randomized prospective trials of mepolizumab in EGPA are currently underway. One study assessed the long-term effectiveness of mepolizumab (at a dose of 300 mg) in patients with EGPA who required oral CS at a dose of ≥5 mg/day of prednisolone equivalent to control their symptoms (NCT 03298061). The second trial evaluated the efficacy of mepolizumab as a remission-inducing agent (at a dose of 300 mg) in comparison to the conventional therapeutic strategy guided by FFS (NCT 05030155).

Given the encouraging results of mepolizumab, other anti-IL5 therapies have also been investigated. These include reslizumab and benralizumab, which are anti-IL-5α receptors. Both were investigated in phase 2 open-label trials with a small number of patients, and the results were promising (206, 207). The efficacy and safety of benralizumab are currently evaluated in comparison to mepolizumab in patients with EGPA receiving standard care therapy (NCT 04157348). The other investigated agent was depemokimab, a long-acting (administered every 26 weeks at a dose of 200 mg) anti-IL-5α receptor drug (NCT05263934).

Since the Th2-pathway activation plays an important role in the pathogenesis of EGPA, drugs studied in asthma may open new possibilities for EGPA treatment. Dupilumab is a humanized monoclonal antibody to the IL-4α receptor that inhibits both IL-4 and IL-13 signaling (208) and is currently approved for moderate and severe uncontrolled asthma (209, 210), CRNP, and atopic dermatitis (21). Another promising drug is itepekimab, an anti-IL-33 monoclonal antibody. In the 2nd phase of a randomized trial, it led to a greater reduction in the mean blood eosinophil count, a lower incidence of asthma exacerbations, and improved lung function in patients with moderate-to-severe asthma with a good safety profile (211). Tezepelumab is the next most recently approved biologic drug (in December 2021) in the US for severe asthma, regardless of its phenotype or biomarkers. This human monoclonal antibody specifically binds to TSLP, preventing it from binding to its heterodimeric receptor (212). Blocking TSLP results in strong inhibition of the CCL2 -related eosinophilic pathway, as well as Th2- related cytokines (IL-4 and 13) and Th17 (212), all of which are involved in the pathogenesis of EGPA (16, 24, 44). Dexpramipexole is an orally bioavailable synthetic aminobenzothiazole that depletes eosinophils by inhibiting their maturation. In a phase 2 trial evaluating the effect of dexpramipexole in moderate-to-severe eosinophilic asthma, its administration led to a lowering of the absolute eosinophil count and improved the forced expiratory volume in 1 sec (FEV1) (213). The results of dexpramipexole in HES are also promising (214).

#### 6.2.2. Anti-CD20 therapy

Rituximab (RTX) is a chimeric monoclonal antibody targeting the CD20 antigen present on B cells, resulting in its depletion (198). Clinical trials showed its effectiveness and safety in GPA and MPA both in the induction (215) and maintenance phases (216). Increasing experience in the treatment of other AAVs with RTX has also led to its use in EGPA. Several case reports and open-label studies have reported the efficacy of RTX in patients with EGPA (46, 217, 218). Recently published data from a retrospective European Collaborative Study involving patients with relapsing and/or refractory disease showed that in those receiving RTX (*N* = 63), the BVAS declined both at 6 and 12 months, and the frequency of remission, partial response, treatment failure, and stopping treatment due to adverse events was 49, 24, 24, and 3%, respectively, without statistically significant differences between ANCA-positive and ANCA-negative patients (219). In 2020, a randomized controlled trial (REOVAS) evaluating the efficacy and safety of RTX in comparison with conventional therapy for remission induction in EGPA was completed (220). In this study, patients with an FFS ≥ 1 (42/105) were randomized to receive CS and RTX/CYC for remission induction, followed by AZA for remission maintenance in both groups. Patients with FFS = 0 (63/105) were randomized to receive RTX with CS or CS as monotherapy. Remission rates for RTX and conventional treatment on days 180 and 360 were comparable in both groups (63.5 vs. 60.4%, and 59.6 vs. 64.2%, respectively). Similarly, the mean duration of remission, relapse rates, and cumulative dose of prednisone was also comparable (220). The study showed that RTX was not superior to the conventional therapeutic strategy to induce vasculitis remission, however, it also did not show that it was inferior to standard therapy. Randomized trial investigating RTX in maintenance therapy compared with standard treatment is underway (MAINRITSEG; NCT 03164473). FVSG experts do not recommend using RTX as first-line induction therapy for EGPA, however, it can be considered for second-line-or-later treatment of severe refractory or relapsed disease, especially following CYC failure (200), at the dosage recommended for GPA and MPA (to induce remission, 375 mg/m^2^ infused once a week for 4 weeks, or 1,000 mg twice at a 15-day interval, which is equally effective and safe; to maintain remission, renewed 500 mg infusions at 6-month intervals for at least 18 months) (200). The ACR guidelines are more flexible. According to these recommendations, RTX can be used in the first line of treatment on par with CYC (the choice is up to the physician), although it is especially preferred in patients with ANCA-positivity and glomerulonephritis. Among those with ANCA-negativity, heart involvement, GI, or severe nervous system involvement, CYC should be considered (200). The safety profile of RTX in patients with EGPA is similar to that of other AAVs, although some studies have reported more frequent allergic reactions to RTX infusion (217). However, recently, the case of non-ischemic cardiomyopathy following RTX treatment has been described (221), which suggests that RTX should be carefully used in case of heart failure, especially in patients with a previous history of cardiac disease. Interestingly, RTX in EGPA has been demonstrated to reduce the production of IL-5, probably by inhibiting B- to T-cell crosstalk (222). Some case series reports have shown the efficacy of RTX for asthma control in EGPA (223).

Recently, there have been reports on the effectiveness of a regimen based on sequential RTX and mepolizumab for the control of EPGA (224, 225). Bettiol et al. (226) published results of the European multicentre retrospective observational study and showed that sequential RTX and mepolizumab treatment (at a dose of 100 mg monthly) is effective to induce and maintain remission of both systemic and respiratory EGPA symptoms. These results seem to support the hypothesis that combining treatments with complementary mechanisms of action might lead to remission of both EGPA components.

#### 6.2.3. Anti-IgE

Omalizumab is a monoclonal antibody that specifically binds to circulating IgE and blocks the inflammatory cascade, notably cell degranulation (mainly basophils and mastocytes), which causes a transient lowering of eosinophilia. It is currently used for the treatment of severe asthma with elevated IgE levels, chronic urticaria, and allergic rhinitis (198). In EGPA, data on the use of omalizumab are inconsistent and scarce and mostly come from case or case series reports. Some of them support the successful use of omalizumab as an adjunct therapy in EGPA patients with severe CS-resistant asthma, but not in those with extrapulmonary manifestations (227, 228). The results of a study comparing the efficacy of biologics in EGPA (RTX, mepolizumab, and omalizumab) showed that omalizumab was associated with significantly lower remission rates (15 vs. 78%) and significantly higher treatment failure (48 vs. 8%) than mepolizumab (219). Information on omalizumab efficacy against vasculitic features in EGPA is lacking, however, two life-threatening cases have been described, who were unresponsive to IS and eventually responded to omalizumab (229). In contrast, a relationship between omalizumab treatment and EGPA development has been described (230). Omalizumab is not recommended as a remission induction therapy for patients with EGPA. It may be considered only in patients who fail or are intolerant to conventional treatment and mepolizumab (200).

### 6.3. Other therapies

Other therapies include interferon α (IFNα), intravenous immunoglobulin (IVIg), and plasmapheresis. Data on the use of IFNα in EGPA are scarce and come mainly from case reports, suggesting that IFNα can induce EGPA remission (231). However, numerous adverse events (e.g., flu-like symptoms, cytopenia, hepatic toxicity, polyneuropathy, and depression) limit their use. Intravenous immunoglobulins may be used off-label in severe cases of refractory AAV in combination with other specific treatments, particularly in patients with severe infectious complications and secondary symptomatic immunological deficits (187). A multicenter double-blind trial showed the efficacy of IVIg as second-line therapy, especially in patients with neural involvement and residual peripheral neuropathy (232). A case of IVIg effectiveness in cardiac involvement in a patient with EGPA not responding to CYC has also been described (233). Regarding plasmapheresis, most data were based on studies that excluded EGPA. Recently published results of a large PEXOVAS study (involving GPA and MPA) showed that the use of plasmapheresis in patients with severe AAV did not reduce the incidence of death or end-stage kidney disease (234). Similar results were presented in a small prospective randomized study of 14 patients with EGPA showing any benefit in adding plasmapheresis to ongoing therapy (235).

## 7. Therapy of asthma

Therapy for asthma is indispensable in the treatment of patients with EGPA and does not differ from the treatment of asthma in the general population (190). Treatment should be adjusted to asthma severity using a stepwise pharmacological approach according to the current International Global Initiative for Asthma (GINA) recommendations (236). Anti-leukotriene drugs are not contraindicated if needed, however, patients require careful follow-up. The scheme of asthma management to control symptoms shows Table 9.

**TABLE 9:** The scheme of asthma management to control symptoms according to GINA 2022.

Tracks of treatment	Step 1 symptoms less than 4–5 days a week	Step 2 symptoms less than 4-5 days a week	Step 3 symptoms most days or waking with asthma once a week or more	Step 4 daily symptoms or waking with asthma once a week or more and low lung function	Step 5 persistent symptoms and/or exacerbations despite optimized treatment with high dose controller medications (usually a high dose of ICS + LABA)
CONTROLLER and RELIEVER: As-needed low dose ICS-formoterol (PREFERRED RELIEVER)	As-needed low dose ICS-formoterol		Low dose maintenance ICS-formoterol	Medium dose maintenance ICS-formoterol	Add-in LAMA Refer for assessment of phenotype Consider high dose maintenance ICS-formoterol, ± anti-IgE, anti-IL5/5R, anti-IL4R, anti-TSLP
CONTROLLER and RELIEVER: As-needed short-acting beta_2_ agonist (ALTERNATIVE RELIEVER)	Take ICS whenever SABA taken	Low dose maintenance ICS	Low-dose maintenance ICS-LABA	Medium dose maintenance ICS-LABA	Add-in LAMA Refer for assessment of phenotype Consider high dose maintenance ICS-LABA, ± anti-IgE, anti-IL5/5R, anti-IL4R, anti-TSLP
Other controller options (less evidence for efficacy or safety, limited indications)		Low dose ICS whenever SABA taken or daily LTRA or HDM SLIT	Medium dose ICS or LTRA or HDM SLIT	Add LAMA or LTRA or HDM SLIT or switch to high dose ICS	Add azithromycin (three times per week) or LTRA; as last resort consider low dose oral CS but consider side effects
	Therapeutic education, skill training, and non-pharmacological treatment				

CS, corticosteroid; ICS, inhaled corticosteroid; LAMA, long-acting muscarinic antagonist; LABA, long-acting beta_2_ agonist; SABA, short-acting beta_2_ agonist; LTRA, leukotriene receptor antagonist; HDM SLIT, house dust mite sublingual immunotherapy; anti-IgE, anti-immunoglobulin E; anti-IL5/5R, anti-interleukin 5/interleukin 5-receptor; anti-IL4R, anti-interleukin 4-receptor; anti-TSLP, anti-cytokine thymic stromal lymphopoietin.

## 8. Therapy for ENT manifestations

Management of ENT manifestations in EGPA can be challenging. The main effective therapy remain intranasal CS. In addition, multiple daily nasal rinses with normal saline may provide some benefit (237, 238). Other drugs, such antihistamines (for proven allergy) or long-term treatment of macrolides, can be tried in cases of intranasal CS inefficacy (190). In the last years, biologic treatment options have been proposed for ENT manifestations. Mepolizumab at a dose of 100 mg monthly has been approved by the FDA in 2021 as an add-on treatment option to standard of care in patients with CRSwNP. The approval has been based on data from the randomized trial (SYNAPSE) which explored the effect of mepolizumab vs. placebo in over 400 patients with CRSwNP, and showed that mepolizumab treatment significantly improved nasal polyp size and nasal obstruction; in addition, in the treated group there was a 57% reduction in the proportion of patients who had surgery compared with placebo group (239). In patients with EGPA, the MIRRA trial demonstrated that mepolizumab significantly reduced the frequency of disease relapses, including both asthma and sinonasal relapses (23).

Regarding to surgical treatment, the role of functional endoscopic sinus surgery (FESS) in EGPA is still a matter of debate. The results of the systematic review advise against FESS as a first-line treatment in EGPA and instead recommends a trial of maximal medication which is often successful in the initial treatment of nasal polyps (240). Surgical removal of nasal polyps can provide transient symptomatic relief but polyp recurrence is frequent (237). In the future, surgery in EGPA will probably collide with the introduction of new biologic drugs in the treatment regimens (238).

## 9. Prognosis and outcomes

Eosinophilic granulomatosis with polyangiitis is considered a milder form of AAV, with lower mortality compared to other AAVs (5, 95). It is now viewed as a chronic disease rather than a fatal condition due to the significant improvement in survival as a result of effective treatment based on CS and/or IS (55). In a monocentric study of 150 patients with EGPA, the 10-year survival rate was 89%, resulting in mortality comparable to that of the general population (82), while in others, overall survival reached 93% after a median follow-up of 6 years (100) and 90% at 7 years (54). However, the prognoses of patients with EGPA differ depending on the presence or absence of prognostic factors (FFS). In addition, relapses, asthma/ENT flares, and disease-related organ damage (sequelae) may severely impair the quality of life of patients with EGPA (54, 100).

Relapses in EGPA remain a major challenge. Their frequency rates vary between 25 and 49% (54, 55, 241). The factors predictive of relapse are not well established and are still under debate. Some studies have shown that peripheral eosinophil count at diagnosis (<3,000 cells/mm^3^), cutaneous manifestation, and positive MPO-ANCA are associated with a higher risk of relapse (54, 55). In others, high IgE levels at onset (101) and FFS > 1 were predictive of relapse (242). Recently, the FCGR3B polymorphism was described as a predictive factor for relapse among EGPA patients with ANCA positivity (243). As serial ANCA monitoring can have some utility in predicting relapses in GPA and MPA, EGPA data are limited because most studies did not provide complete results on repeat ANCA testing following treatment (244). However, according to the European EGPA Study Group recommendations, repeat ANCA testing is indicated in patients with MPO-ANCA-positive EGPA because persistence, rise, or reappearance of ANCA may justify more frequent clinical assessments (152).

The next problems in patients with EGPA are asthma and/or ENT flares, which are mostly independent of disease activity (82, 100). Although not life-threatening, both significantly contribute to patient morbidity, which is associated with persistent symptoms and exposure to long-term CS therapy and its associated adverse events (245). Among a multicenter cohort of 101 patients with EGPA with a median follow-up of 6 years, 92.5% still received systemic CS at the end of the study (100). In an American study involving 354 patients with a median follow-up of 7 years, at the last study visit, only 12.6% had been off all therapies for more than 2 years during their follow-up (241). While vasculitis relapses tend to ensue within the first 2 years following diagnosis, asthma and ENT manifestations tend to persist or relapse long after vasculitis has resolved (246). Sequeales are observed in 80% of patients with EGPA, regardless of the initial severity of the disease (54, 100). Next to asthma and ENT symptoms, it includes persistent polyneuropathy (45%), osteoporosis (30%), severe lung disease (17%), chronic kidney disease (13%), and chronic heart failure (11%) (54, 100).

A recent study from the US (247) excellently reflects the high burden of EGPA. The results showed that all-cause healthcare costs were 2.5-fold higher in patients with EGPA than in those with asthma alone (with similar geographic and insurance status). Furthermore, all-cause healthcare resource utilization and use of systemic CS were also significantly greater in EGPA, with more than one-third of these patients experiencing relapses (247). On the other hand, although the burden of the disease remains still high, the mortality and morbidity of patients have essentially decreased due to the change in approach toward the treatment over the years. A Spanish study analyzing the outcomes of AAV patients (including EGPA) showed improved results with a significant decrease in mortality and treatment-related morbidity in patients diagnosed after 2000 compared to those diagnosed prior to 2000, which was related to the use of less toxic regimens adapted to the disease activity and stage, and a drastic reduction in the cumulative CYC and CS dose (113). This trend in treatment continues with the implementation and search for new biologic therapies.

## 10. Conclusion

Despite notable progress in the understanding of its pathogenesis and disease management, EGPA remains a major diagnostic and therapeutic challenge. Its dual categorization with HESs and systemic vasculitides leads to varied clinical presentation, which requires careful differentiation from other mimicking disorders. To date, there are no universally approved diagnostic criteria, and diagnosis remains mainly clinical. The role of ANCA is not fully understood; but two phenotypes have been defined according to ANCA status, with consistently different genetic backgrounds, manifestations, prognoses, and treatment responses. However, ANCA is present only in approximately 30–40% of patients, and there is still an ongoing debate over whether EGPA should be recognized in cases without the presence of vasculitis and ANCA. Breakthroughs in clinical practice are novel classification criteria that are expected to accelerate clinical studies on EGPA in the future. Although the prognosis is good, relapses in EGPA are frequent, and many patients have chronic symptoms that require long-term treatment with CS. Although effective, conventional therapy is not satisfactory for relapse prevention and resolution of chronic symptoms and is burdened with high toxicity. In this context, new biological agents are a valid therapeutic alternative, although more data are required.

## Author contributions

JF and ER contributed to the conception and design of the work and drafted the manuscript. Both authors approved the final version of the manuscript.

## References

[1] White J Dubey S. Eosinophilic granulomatosis with polyangiitis: a review. *Autoimmun Rev.* (2022) 22:103219. 10.1016/j.autrev.2022.103219 36283646

[2] Churg J Strauss L. Allergic granulomatosis, allergic angiitis, and periarteritis nodosa. *Am J Pathol.* (1951) 27:277–301. 14819261 PMC1937314

[3] Springer J Kalot M Husainat N Byram K Dua A James K Eosinophilic granulomatosis with polyangiitis: a systematic review and meta-analysis of test accuracy and benefits and harms of common treatments. *ACR Open Rheumatol.* (2021) 3:101–10. 10.1002/acr2.11194 33512787 PMC7882521

[4] Harold L Patterson M Andrade S Dube T Go A Buist A Asthma drug use and the development of Churg-Strauss syndrome (CSS). *Pharmacoepidemiol Drug Saf.* (2007) 16:620–6. 10.1002/pds.1353 17192840

[5] Nguyen Y Guillevin L. Eosinophilic granulomatosis with polyangiitis (Churg-Strauss). *Semin Respir Crit Care Med.* (2018) 39:471–81. 10.1055/s-0038-1669454 30404114

[6] Jariwala M Laxer R. Childhood GPA, EGPA, and MPA. *Clin Immunol.* (2020) 211:108325. 10.1016/j.clim.2019.108325 31837445

[7] Fijolek J Wiatr E Piotrowska-Kownacka D Roszkowski-Sliz K. The role of peripheral eosinophilia in diagnosing lung disorders: experience from a single pneumonological center. *Multidiscip Respir Med.* (2021) 16:770. 10.4081/mrm.2021.770 34858593 PMC8581820

[8] Jennette J Falk R Bacon P Basu N Cid M Ferrario F 2012 revised international Chapel Hill consensus conference nomenclature of vasculitides. *Arthritis Rheum.* (2012) 65:1–11. 10.1002/art.37715 23045170

[9] Sinico R Di Toma L Maggiore U Bottero P Radice A Tosoni C Prevalence and clinical significance of antineutrophil cytoplasmic antibodies in Churg-Strauss syndrome. *Arthritis Rheum.* (2005) 52:2926–35. 10.1002/art.21250 16142760

[10] Sable-Fourtassou R Cohen P Mahr A Pagnoux C Mouthon L Jayne D Antineutrophil cytoplasmic antibodies and Churg-Strauss syndrome. *Ann Intern Med.* (2005) 143:632–8. 10.7326/0003-4819-143-9-200511010-00006 16263885

[11] Valent P Klion A Horny H Roufosse F Gotlib J. Contemporary consensus proposal on criteria and classification of eosinophilic disorders and related syndromes. *J Allergy Clin Immunol.* (2012) 130:607.e–12.e. 10.1016/j.jaci.2012.02.019 22460074 PMC4091810

[12] Egan A Kronbichler A Neumann I Bettiol A Carlson N Cid M The sound of interconnectivity; the European vasculitis society 2022 report. *Kidney Int Rep.* (2022) 7:1745–57. 10.1016/j.ekir.2022.05.018 35967106 PMC9366365

[13] Jennette JC Falk R Andrassy K Bacon P Churg J Gross W Nomenclature of systemic vasculitides. Proposal of an international consensus conference. *Arthritis Rheum.* (1994) 37:187–92. 10.1002/art.1780370206 8129773

[14] Cottin V Bel E Bottero P Dalhoff K Humbert M Lazor R Revisiting the systemic vasculitis in eosinophilic granulomatosis with polyangiitis (Churg-Strauss). A study of 157 patients by the Groupe d’Etudes et de Recherche sur les Maladies Orphelines Pulmonaires and the European respiratory society taskforce on eosinophilic granulomatosis with polyangiitis (Churg-Strauss). *Autoimmun Rev.* (2017) 16:1–9. 10.1016/j.autrev.2016.09.018 27671089

[15] Fagni F Bello F Emmi G. Eosinophilic granulomatosis with polyangiitis: dissecting the pathophysiology. *Front Med.* (2021) 8:627776. 10.3389/fmed.2021.627776 33718405 PMC7943470

[16] Trivioli G Terrier B Vaglio A. Eosinophilic granulomatosis with polyangiitis: understanding the disease and its management. *Rheumatology.* (2020) 59:iii84–94. 10.1093/rheumatology/kez570 32348510

[17] Dejaco C Oppl B Monach P Cuthbertson D Carette S Hoffman G Serum biomarkers in patients with relapsing eosinophilic granulomatosis with polyangiitis (Churg-Strauss). *PLoS One.* (2015) 10:e0121737. 10.1371/journal.pone.0121737 25812008 PMC4374913

[18] Kiene M Csernok E Muller A Metzler C Trabandt A Gross W. Elevated interleukin-4 and interleukin-13 production by T cell lines from patients with Churg-Strauss syndrome. *Arthritis Rheum.* (2001) 44:469–73. 10.1002/1529-0131(200102)44:23.0CO;2-0 11229479

[19] Jakiela B Szczeklik W Plutecka H Sokolowska B Mastalerz L Sanak M Increased production of IL-5 and dominant Th2-type response in airways of Churg-Strauss syndrome patients. *Rheumatology.* (2012) 51:1887–93. 10.1093/rheumatology/kes171 22772323

[20] Gioffredi A Maritati F Oliva E Buzio C. Eosinophilic granulomatosis with polyangiitis: an overview. *Front Immunol.* (2014) 5:549. 10.3389/fimmu.2014.00549 25404930 PMC4217511

[21] Nagase H Ueki S Fujieda S. The roles of IL-5 and anti-IL-5 treatment in eosinophilic diseases: asthma, eosinophilic granulomatosis with polyangiitis, and eosinophilic chronic rhinosinusitis. *Allergology Int.* (2020) 69:178–86. 10.1016/j.alit.2020.02.002 32139163

[22] Eng S DeFelice M. The role and immunobiology of eosinophils in the respiratory system: a comprehensive review. *Clinic Rev Allergy Immunol.* (2016) 50:140–58. 10.1007/s12016-015-8526-3 26797962

[23] Wechsler M Akuthota P Jayne D Khoury P Klion A Langford C Mepolizumab or placebo for eosinophilic granulomatosis with polyangiitis. *N Engl J Med.* (2017) 376:1921–32. 10.1056/NEJMoa1702079 28514601 PMC5548295

[24] Louten J Boniface K de Waal Malefyt R. Development and function of Th17 cells in health and disease. *J Allergy Clin Immunol.* (2009) 123:1004–11. 10.1016/j.jaci.2009.04.003 19410689

[25] Jakiela B Sanak M Szczeklik W Sokolowska B Plutecka H Mastalerz L Both Th2 and Th17 responses are involved in the pathogenesis of Churg-Strauss syndrome. *Clin Exp Rheumatol.* (2011) 29 (suppl.64):S23–34. 21470488

[26] Khoury P Grayson P Klion A. Eosinophils in vasculitis: characteristics and roles in pathogenesis. *Nat Rev Rheumatol.* (2014) 10:474–83. 10.1038/nrrheum.2014.98 25003763 PMC4849122

[27] Fujioka A Yamamoto T Takasu H Kawano K Masuzawa M Katsuoka K The analysis of mRNA expression of cytokines from skin lesions in Churg-Strauss syndrome. *J Dermatol.* (1998) 25:171–7. 10.1111/j.1346-8138.1998.tb02375.x 9575680

[28] Ebina-Shibuya R Leonard W. Role of thymic stromal lymphopoietin in allergy and beyond. *Nat Rev Immunol.* (2022) 1:1–14. 10.1038/s41577-022-00735-y 35650271 PMC9157039

[29] Boita M Guida G Circosta P Elia A Stella S Heffler E The molecular and functional characterization of clonally expanded CD8+ TCR BV T cells in eosinophilic granulomatosis with polyangiitis (EGPA). *Clin Immunol.* (2014) 152:152–63. 10.1016/j.clim.2014.03.001 24632064

[30] Drage L Davis M De Castro F Van Keulen V Weiss E Gleich G Evidence for pathogenic involvement of eosinophils and neutrophils in Churg-Strauss syndrome. *J Am Acad Dermatol.* (2002) 47:209–16. 10.1067/mjd.2002.124600 12140466

[31] Arima M Kanoh T. Eosinophilic myocarditis associated with dense deposits of eosinophil cationic protein (ECP) in endomyocardium with high serum ECP. *Heart.* (1999) 81:669–75. 10.1136/hrt.81.6.669 10336931 PMC1729068

[32] Fettrelet T Gigon L Karaulov A Yousefi S Simon H. The enigma of eosinophil degranulation. *Int J Mol Sci.* (2021) 22:7091. 10.3390/ojms22137091 PMC8268949 34209362

[33] Koike H Nishi R Furukawa S Mouri N Fukami Y Iijima M In vivo visualization of eosinophil secretion in eosinophilic granulomatosis with polyangiitis: an ultrastructural study. *Allergol Int.* (2022) 71:373–82. 10.1016/j.alit.2022.02.009 35428588

[34] Vega Villanueva K Espinoza L. Eosinophilic vasculitis. *Cur Rheumatol Rep.* (2020) 22:5. 10.1007/s11926-020-0881-2 31927633

[35] Emmi G Silvestri E Squatrito D Amedei A Niccolai E D’Elios M Thrombosis in vasculitis: from pathogenesis to treatment. *Thrombosis J.* (2015) 13:15. 10.1186/s12959-015-0047-z 25883536 PMC4399148

[36] Noguchi H Kephart G Colby T Gleich G. Tissue eosinophilia and eosinophil degranulation in syndromes associated with fibrosis. *Am J Pathol.* (1992) 140:521–8. 1739138 PMC1886427

[37] Filley W Holley K Kephart G Gleich G. Identification by immunofluorescence of eosinophil granule major basic protein in lung tissues of patients with bronchial asthma. *Lancet.* (1982) 2:11–6. 10.1016/S0140-6736(82)91152-7 6177986

[38] Terrier B Bieche I Maisonobe T Laurendeau I Rosenzwajg M Kahn J Interleukin -25: a cytokine linking eosinophils and adaptive immunity in Churg-Strauss syndrome. *Blood.* (2010) 16:4523–31. 10.1182/blood-2010-02-267542 20729468

[39] Moore P Church T Chism D Panettieri R Jr Shore S. IL-13 and IL-4 cause eotaxin release in human airway smooth muscle cells: a role for ERK. *Am J Physiol Lung Cell Mol Physiol.* (2002) 282:L847–53. 10.1152/ajplung.00245.2001 11880312

[40] Polzer K Karonitsch T Neumann T Eger G Haberler C Soleiman A Eotaxin-3 is involved in Churg-Strauss syndrome – a serum marker closely correlating with disease activity. *Rheumatology.* (2008) 47:804–8. 10.1093/rheumatology/ken033 18397958

[41] Zwerina J Bach C Martorana D Jatzwauk M Hegasy G Moosig F Eotaxin-3 in Churg-Strauss syndrome: a clinical and immunogenetic study. *Rheumatology.* (2011) 50:1823–7. 10.1093/rheumatology/keq445 21266446

[42] Emmi G Silvestri E Marconi R Carrai V Fanelli T Zucchini P First report of FIP1L1-PDGFRalpha-positive eosinophilic granulomatosis with polyangiitis. *Rheumatology.* (2015) 54:1751–3. 10.1093/rheumatology/kev242 26106208

[43] Triggianese P D’Antonio A Conigliaro P Buccisano F Fonti G Chimenti M A new focus on thyrosine kinases inhibitors in eosinophilic granulomatosis with polyangiitis. *Clin Exp Rheumatol.* (2021) 39 (suppl.129):S180–1. 10.55563/clinexprheumatol/rrjt9m 33338003

[44] Beketova T Volkov M Naryshkin E Novoselova T Nasonov E. Imatinib mesylate use in refractory eosinophilic granulomatosis with polyangiitis: a literature review and a case report. *Clin Rheumatol.* (2018) 37:1729–35. 10.1007/s10067-018-4018-1 29564565

[45] Tsurikisawa N Oshikata C Watanabe M Tsuburai T Kaneko T Saito H. Innate immune response refelcts disease activity in eosinophilic granulomatosis with polyangiitis. *Clin Exp Allergy.* (2018) 48:1305–16. 10.1111/cea.13209 29908086

[46] Akiyama M Kaneko Y Takeuchi T. Rituximab for the treatmet of eosinophilic granulomatosis with polyangiitis: a systematic literature review. *Autoimmun Rev.* (2021) 20:102737. 10.1016/j.autrev.2020.102737 33340770

[47] Wu Z Zhang S Li P Song N Zhang F Li Y. Elevated serum IgG4 was found in eosinophilic granulomatosis with polyangiitis. *J Clin Rheumatol.* (2021) 27:e501–4. 10.1097/RHU.0000000000001606 33315788

[48] Vaglio A Strehl J Manger B Maritati F Alberici F Beyer C IgG4 immune response in Churg-Strauss syndrome. *Ann Rheum Dis.* (2012) 71:390–3. 10.1136/ard.2011.155382 22121132

[49] Tsurikisawa N Saito H Oshikata C Tsuburai T Akiyama K. Decreases in the numbers of peripheral blood regulatory T cells, and increases in the levels of memory and activated B cells in patients with active eosinophilic granulomatosis and polyangiitis. *J Clin Immunol.* (2013) 33:965–76. 10.1007/s10875-013-9898-x 23624693

[50] Kubo S Kanda R Nawata A Miyazaki Y Kawabe A Hanami K Eosinophilic granulomatosis with polyangiitis exhibits T cell activation and IgG4 immune response in the tissue; comparison with IgG4-related disease. *RMD Open.* (2022) 8:e002086. 10.1136/rmdopen-2021-002086 35260476 PMC8906049

[51] Shochet L Holdsworth S Kitching A. Animal models of ANCA associated vasculitis. *Front Immunol.* (2020) 11:525. 10.3389/fimmu.2020.00525 32373109 PMC7179669

[52] Schlieben D Korbet S Kimura R Schwartz M Lewis E. Pulmonary-renal syndrome in a newborn with placental transmission of ANCAs. *Am J Kidney Dis.* (2005) 45:758–61. 10.1053/j.ajkd.2005.01.001 15806479

[53] Falk R Terrel R Charles L Jennette J. Anti-neutrophil cytoplasmic antibodies induce neutrophils to degranulate and produce oxygen radicals in vitro. *Proct Natl Acad Sci U.S.A.* (1990) 87:4115–9. 10.1073/pnas.87.11.4115 2161532 PMC54058

[54] Samson M Puechal X Devilliers H Ribi C Cohen P Stern M Long-term outcomes of 118 patients with eosinophilic granulomatosis with polyangiitis (Churg-Strauss syndrome) enrolled in two prospective trials. *J Autoimmun.* (2013) 43:60–9. 10.1016/j.jaut.2013.03.003 23590801

[55] Comarmond C Pagnoux C Khellaf M Cordier J Hamidou M Viallard J Eosinophilic granulomatosis with polyangiitis (Churg-Strauss). Clinical characteristics and long-term followup of the 383 patients enrolled in the French vasculitis study group Cohort. *Arthritis Rheum.* (2013) 65:270–81. 10.1002/art.37721 23044708

[56] Lyons P Peters J Alberici F Liley J Coulson R Astle W Genome-wide association study of eosinophilic granulomatosis with polyangiitis reveals genomic loci stratified by ANCA status. *Nat Comm.* (2019) 10:5120. 10.1038/s41467-019-12515-9 31719529 PMC6851141

[57] Chaigne B Terrier B Thieblemont N Witko-Sarsat V Mouthon L. Dividing the Janus vasculitis? Pathophysiology of eosinophilic granulomatosis with polyangiitis. *Autoimmun Rev.* (2016) 15:139–45. 10.1016/j.autrey.2015.10.006 26506114

[58] Brinkmann V Reichard U Goosmann C Fauler B Uhlemann Y Weiss D Neutrophil extracellular traps kill bacteria. *Science.* (2004) 303:1532–5. 10.1126/science.1092385 15001782

[59] Arneth B Arneth R. Neutrophil extracellular traps (NETs) and vasculitis. *In J Med Sci.* (2021) 18:1532–40. 10.7150/ijms.53728 33746569 PMC7976562

[60] Sciascia S Ponticelli C Roccatello D. Pathogenesis-based new perspectives of management of ANCA-associated vasculitis. *Autoimmun Rev.* (2022) 21:103030. 10.1016/j.autrev.2021.103030 34971805

[61] Sangaletti S Tripodo C Chiodoni C Guarnotta C Cappetti B Casalini P Neutrophil extracellular traps mediate transfer of cytoplasmic neutrophil antigens to myeloid dendritic cells toward ANCA induction and associated autoimmunity. *Blood.* (2012) 120:3007–18. 10.1182/blood-2012-03-416156 22932797

[62] Fukuchi M Kamide Y Ueki S Miyabe Y Konno Y Oka N Eosinophil ETosis-mediated release of galectin-10 in eosinophilic granulomatosis with polyangiitis. *Arthritis Rheumatol.* (2021) 73:1683–93. 10.1002/art.41727 33750029 PMC8403105

[63] Mukherjee M Thomas S Radford K Dvorkin-Gheva A Davydchenko S Kjarsgaard M Sputum antineutrophil cytoplasmic antibodies in serum antineutrophil cytoplasmic antibody-negative eosinophilic granulomatosis with polyangiitis. *Am J Respir Crit Care Med.* (2019) 199:158–70. 10.1164/rccm.201804-0809OC 30179583

[64] Hashimoto T Ueki S Kamide Y Miyabe Y Fukuchi M Yokoyama Y Increased circulating cell-free DNA in eosinophilic granulomatosis with polyangiitis: implications for eosinophil extracellular traps and immunothrombosis. *Front Immunol.* (2022) 12:801897. 10.3389/fimmu.2021.801897 35095884 PMC8790570

[65] Wieczorek S Hellmich B Gross W Epplen J. Associations of Churg-Strauss syndrome with the HLA-DRB1 locus, and relationship to the genetics of antineutrophil cytoplasmic antibody-associated vasculitides: comment on the article by Vaglio et al. *Arthritis Rheum.* (2008) 58:329–30. 10.1002/art.23209 18163478

[66] Vaglio A Martorana D Maggiore U Grasselli C Zanetti A Pesci A HLA-DRB4 as a genetic risk factor for Churg-Strauss syndrome. *Arthritis Rheum.* (2007) 56:3159–66. 10.1002/art.22834 17763415

[67] Wieczorek S Hellmich B Arning L Moosig F Lamprecht P Gross W Functionally relevant variations of the interleukin-10 gene associated with antineutrophil cytoplasmic antibody-negative Churg-Strauss syndrome, but not with Wegener’s granulomatosis. *Arthritis Rheum.* (2008) 58:1839–48. 10.1002/art.23496 18512809

[68] Mouthon L Khaled M Cohen P Guillevin L Mouthon L Subra J. Systemic small sized vessel vasculitis after massive antigen inhalation. *Ann Rheum Dis.* (2001) 60:1288–94. PMC1753838 11534512

[69] Guillevin L Amouroux J Arbeille B Boura R. Churg-Strauss angiitis. Arguments favoring the responsibility of inhaled antigens. *Chest.* (1991) 100:1472–3. 10.1378/chest.100.5.1472 1935321

[70] Harada M Imokawa S Miwa S Nihashi F Aono Y Amano Y Chronic pulmonary aspergillosis may cause eosinophilic granulomatosis with polyangiitis via allergic bronchopulmonary aspergillosis. *Oxf Med Case Rep.* (2019) 19:omy126. 10.1093/omcr/omy126 30800324 PMC6380532

[71] Coutinho I Lopes M Lima F Ventura C Rabadao E Alfaro T Concomitant allergic bronchopulmonary aspergillosis and eosinophilic granulomatosis with polyangiitis after *Aspergillus niger* infection. *Pulmonology.* (2022) 28:231–4. 10.1016/j.pulmoe.2021.12.004 35361562

[72] Karampoor S Afrashteh F Rahmani S Laali A. Eosinophilic granulomatosis with polyangiitis after COVID-19: a case report. *Respir Med Case Rep.* (2022) 38:101702. 10.1016/j.rmer.2022.101702 PMC9279185 35854792

[73] Wechsler M Finn D Guanawardena D Westlake R Barker A Haranath S Churg-Strauss syndrome in patients receiving montelukast as treatment for asthma. *Chest.* (2000) 117:708–13. 10.1378/chest.117.3.708 10712995

[74] Wechsler M Wong D Miller M Lawrence-Miyasaki L. Churg-Strauss syndrome in patients treated with omalizumab. *Chest.* (2009) 136:507–18. 10.1378/chest.08-2990 19411292

[75] Hauser T Mahr A Metzler C Coste J Sommerstein R Gross W The leucotriene receptor antagonist montelukast and the risk of Churg-Strauss syndrome: a case-crossover study. *Thorax.* (2008) 63:677–82. 10.1136/thx.2007.087825 18276721

[76] Schroeder J Folci M Losappio L Chevallard M Sinico R Mirone C Anti-neutrophil cytoplasmic antibodies positivity and anti-leukotrienes in eosinophilic granulomatosis with polyangiitis: a retrospective monocentric study on 134 Italian patients. *Int Arch Allergy Immunol.* (2019) 180:64–71. 10.1159/000500544 31189169

[77] Ikeda M Ohshima N Kawashima M Shiina M Kitani M Suzukawa M. Severe asthma where eosinophilic granulomatosis with polyangiitis became apparent after the discontinuation of dupilumab. *Intern Med.* (2022) 61:755–9. 10.2169/internalmedicine.7990-21 34393172 PMC8943368

[78] Lim A Antony A Gingold M Simpson I Looi W MacDonald M. Emergence of extrathoracic manifestations of eosinophilic granulomatosis with polyangiitis during benralizumab treatment. *Rheumatology Adv Prac.* (2021) 5:rkab033. 10.1093/rap/rkab033 34124538 PMC8190011

[79] Bottero P Bonini M Vecchio F Grittini A Patruno G Colombo B The common allergens in the Churg-Strauss syndrome. *Allergy.* (2007) 62:1288–94. 10.1111/j.1398-9995.2007.01486.x 17919144

[80] Nappi E De Santis M Paoletti G Pelaia C Terenghi F Pini D New onset of granulomatosis with polyangiitis following mRNA-based COVID-19 vaccine. *Vaccines (Basel).* (2022) 10:716. 10.3390/vaccines10050716 35632472 PMC9144767

[81] Lanham J Elkon K Pusey C Hughes G. Systemic vasculitis with asthma and eosinophilia: a clinical approach to the Churg-Strauss syndrome. *Medicine (Baltimore).* (1984) 63:65–81. 10.1097/00005792-198403000-00001 6366453

[82] Moosig F Bremer J Hellmich B Holle J Holl-Ulrich K Laudien M A vasculitis centre based management strategy leads to improved outcome in eosinophilic granulomatosis and polyangiitis (Churg-Strauss, EGPA): monocentric experiences in 150 patients. *Ann Rheum Dis.* (2013) 72:1011–7. 10.1136/annrheumdis-2012-201531 22887848

[83] Tsurikisawa N Oshikata C Kinoshita A Tsuburai T Saito H. Longterm prognosis of 121 patients with eosinophilic granulomatosis with polyangiitis in Japan. *J Rheumatol.* (2017) 44:1206–15. 10.3899/jrheum.161436 28572468

[84] Wojcik K Wawrzycka-Adamczyk K Wludarczyk A Sznajd J Zdrojewski Z Masiak A Clinical characteristics of polish patients with ANCA-associated vasculitides-retrospective analysis of POLVAS registry. *Clin Rheumatol.* (2019) 38:2553–63. 10.1007/s10067-019-04538-w 31016580

[85] Healy B Bibby S Steele R Weatherall M Nelson H Beasley R. Antineutrophil cytoplasmic autoantibodies and myeloperoxidase autoantibodies in clinical expression of Churg-Strauss syndrome. *J Allergy Clin Immunol.* (2013) 131:571–6. 10.1016/j.jaci.2012.05.058 22920496

[86] Bettiol A Sinico R Schiavon F Monti S Bozzolo E Franceschini F Risk of acute arterial and venous thromboembolic events in eosinophilic granulomatosis with polyangiitis (Churg-Strauss syndrome). *Eur Respir J.* (2021) 57:2004158. 10.1183/13993003.04158-2020 33833031

[87] Fijolek J Wiatr E Bujnowski P Piotrowska-Kownacka D Roszkowski-Sliz K. Evaluation of prognostic factors for patients with eosinophilic granulomatosis with polyangiitis recruited at the pneumonological centre and mainly ANCA negativity: a retrospective analysis of a single cohort in Poland. *Mod Rheumatol.* (2023) road001. 10.1093/mr/road001 36658715

[88] Berti A Volcheck G Cornec D Smyth R Specks U Keogh K. Severe/uncontrolled asthma and overall survival in atopic patients with eosinophilic granulomatosis with polyangiitis. *Respir Med.* (2018) 142:66–72. 10.1016/j.rmed.2018.07.017 30170804

[89] Cottin C Bel E Bottero P Dalhoff K Humbert M Lazor R Respiratory manifestations of eosinophilic granulomatosis with polyangiitis (Churg-Strauss). *Eur Respir J.* (2016) 48:1429–41. 10.1183/13993003.00097-2016 27587545

[90] Cottin C Khouatra C Dubost R Glerant J Cordier J. Persistent airflow obstruction in asthma of patients with Churg-Strauss syndrome and long-term follow-up. *Allergy.* (2009) 64:589–95. 10.1111/j.1398-9995.2008.01854.x 19154547

[91] Latorre M Baldini C Seccia V Pepe P Novelli F Celi A Asthma control and airway inflammation in patients with eosinophilic granulomatosis with polyangiitis. *J Allergy Clin Immunol Pract.* (2016) 4:512–9. 10.1016/j.jaip.2015.12.014 26883543

[92] Szczeklik W Sokolowska B Zuk J Mastalerz L Szczeklik A Musial J. The course of asthma in Churg-Strauss syndrome. *J Asthma.* (2011) 48:183–7. 10.3109/02770903.2010.551796 21247352

[93] Berti A Cornec D Moura M Smyth R Dagna L Specks U Eosinophilic granulomatosis with polyangiitis. Clinical predictors of long-term asthma severity. *Chest.* (2020) 157:1086–99. 10.1016/j.chest.2019.11.045 31958440

[94] Bacciu A Bacciu S Mercante G Ingegnoli F Grasselli C Vaglio A Ear, nose and throat manifestations of Churg-Strauss syndrome. *Acta Oto-Laryngol.* (2006) 126:503–9. 10.1080/00016480500437435 16698700

[95] Bacciu A Buzio C Giordano D Pasanisi E Vincenti V Mercante G Nasal polyposis in Churg-Strauss syndrome. *Laryngoscope.* (2008) 118:325–9. 10.1097/MLG.0b013e318159889d 17989571

[96] Seccia V Baldini C Latorre M Gelardi M Dallan I Cristofani-Mencacci L Focus on the involvement of the nose and paranasal sinuses in eosinophilic granulomatosis with polyangiitis (Churg-Strauss syndrome): nasal cytology reveals infiltration of eosinophils as a very common feature. *Int Arch Allergy Immunol.* (2018) 175:61–9. 10.1159/000484602 29393242

[97] Brescia G Padoan R Schiavon F Contro G Parrino D Tealdo G Nasal polyps in eosinophilic granulomatosis with polyangiitis: structured histopathology and CD105 expression. *Am J Otolaryngol.* (2020) 41:102661. 10.1016/j.amjoto.2020.102661 32810787

[98] Greco A Rizzo M De Virgilio A Gallo A Fusconi M Ruoppolo G Churg-Strauss syndrome. *Autoimmun Rev.* (2015) 14:341–8. 10.1016/j.autrev.2014.12.004 25500434

[99] Guillevin L Cohen P Gayraud M Lhote F Jarrousse B Casassus P. Churg-Strauss syndrome. Clinical study and long-term follow-up of 96 patients. *Medicine (Baltimore).* (1999) 78:26–37. 10.1097/00005792-199901000-00003 9990352

[100] Durel C Berthiller J Caboni S Jayne D Ninet J Hot A. Long-term followup of a multicenter cohort of 101 patients with eosinophilic granulomatosis with polyangiitis (Churg-Strauss). *Arthritis Care Res.* (2016) 68:374–87. 10.1002/acr.22686 26315340

[101] Saku A Furuta S Hiraguri M Ikeda K Kobayashi Y Kagami SI Longterm outcomes of 188 Japanese patients with eosinophilic granulomatosis with polyangiitis. *J Rheumatol.* (2018) 45:1159–66. 10.3899/jrheum.171352 29907668

[102] Fijolek J Wiatr E Gawryluk D Langfort R Kober J Piotrowska-Kownacka D The basis of Churg-Strauss syndrome diagnosis in own material. *Pneumonol Alergol Pol.* (2012) 80:20–8. 22187177

[103] Bernheim A McLoud T. A review of clinical and imaging findings in eosinophilic lung diseases. *AJR Am J Roentgenol.* (2017) 208:1002–10. 10.2214/AJR.16.17315 28225641

[104] Choi Y Im J Han B Kim J Lee K Myoung N. Thoracic manifestation of Churg-Strauss syndrome. Radiologic and clinical findings. *Chest.* (2000) 117:117–24. 10.1378/chest.117.1.117 10631208

[105] Zimmermann N Wikenheiser-Brokamp K. Hypereosinophilic syndrome in the differential diagnosis of pulmonary infiltrates with eosinophilia. *Ann Allergy Asthma Immunol.* (2018) 121:179–85. 10.1016/j.anai.2018.05.014 29803709

[106] Kim Y Lee K Han J Chong S Chung M Yi C Pulmonary involvement in Churg-Strauss syndrome: an analysis of CT, clinical, and pathologic findings. *Eur Radiol.* (2007) 17:3157–65. 10.1007/s00330-007-0700-4 17605012

[107] Nakamoto K Saraya T Ogawa Y Ishii H Takizawa H. Comparison of findings on thoracic computed tomography with the severity and duration of bronchial asthma in patients with eosinophilic granulomatosis with polyangiitis. *Respir Med.* (2018) 139:101–5. 10.1016/j.rmed.2018.05.003 29857992

[108] Cottin V. Eosinophilic lung diseases. *Clin Chest Med.* (2016) 37:535–56. 10.1016/j.ccm.2016.04.015 27514599

[109] Thompson G Bourne M Jr Moura M Baqir M Cartin-Ceba R Makol A Pleuritis and pericarditis in antineutrophil cytoplasmic autoantibody-associated vasculitis. *Chest.* (2021) 160:572–81. 10.1016/j.chest.2021.02.049 33667492

[110] Pagnoux C Mahr A Cohen P Guillevin L. Presentation and outcome of gastrointestinal involvement in systemic necrotizing vasculitides. Analysis of 62 patients with polyarteritis nodosa, microscopic polyangiitis, Wegener granulomatosis, Churg-Strauss syndrome, or rheumatoid arthritis-associated vasculitis. *Medicine.* (2005) 84:115–28. 10.1097/01.md.0000158825.87055.0b 15758841

[111] Furuta S Iwamoto T Nakajima H. Update on eosinophilic granulomatosis with polyangiitis. *Allergol Int.* (2019) 68:430–6. 10.1016/j.alit.2019.06.004 31266709

[112] Mavrogeni S Karabela G Gialafos E Stavropoulos E Spiliotis G Katsifis G Cardiac involvement in ANCA (+) and ANCA (–) Churg-Strauss syndrome evaluated by cardiovascular magnetic resonance. *Inflamm Allergy Drug Targ.* (2013) 12:322–7. 10.2174/18715281113129990054 23909889

[113] Solans-Laque R Fraile G Rodrigues-Carballeira M Caminal L Castillo M Martinez-Valle F Clinical characteristics and outcome of Spanish patients with ANCA-associated vasculitides. Impact of the vasculitis type, ANCA specificity, and treatment on mortality and morbidity. *Medicine.* (2017) 96:e6083. 10.1097/MD.0000000000006083 28225490 PMC5569416

[114] Guillevin L Pagnoux C Seror R Mahr A Mouthon L Le Toumelin P The Five-Factor Score revisited. Assessment of prognoses of systemic necrotizing vasculitides based on the French Vasculitis Study Group (FVSG) cohort. *Medicine.* (2011) 90:19–27. 10.1097/MD.0b013e18205a4c6 21200183

[115] Misra D Shenoy S. Cardiac involvement in primary systemic vasculitis and potential drug therapies to reduce cardiovascular risk. *Rheumatol Int.* (2017) 37:151–67. 10.1007/s00296-016-3435-1 26886388

[116] Zampieri M Emmi G Beltrami M Fumagalli C Urban M Dei L Cardiac involvement in eosinophilic granulomatosis with polyangiitis (formerly Churg-Strauss syndrome): prospective evaluation at a tertiary referral centre. *Eur J Int Med.* (2021) 85:68–79. 10.1016/j.ejim.2020.12.008 33358337

[117] Neumann T Manger B Schmid M Kroegel C Hansch A Reinhardt D Cardiac involvement in Churg-Strauss syndrome. Impact of myocarditis. *Medicine.* (2009) 88:236–43. 10.1097/MD.0b013e3181af35a5 19593229

[118] Garcia-Vives E Rodrigues-Palomares J Harty L Solans-Laque R Jayne D. Heart disease in eosinophilic granulomatosis with polyangiitis (EGPA) patients: a screening approach proposal. *Rheumatology.* (2021) 60:4538–47. 10.1093/rheumatology/keab027 33493294

[119] Seguela P Iriart X Acar P Montaudon M Roudaut R Thambo J. Eosinophilic cardiac disease: molecular, clinical and imaging aspects. *Arch Cardiovasc Dis.* (2015) 108:258–68. 10.1016/j.acvd.2015.01.006 25858537

[120] Hazebroek M Kemna M Schalla S Sanders-van Wijk S Gerretsen SC Dennert R Prevalence and prognostic relevance of cardiac involvement in ANCA-associated vasculitis: eosinophilic granulomatosis with polyangiitis and granulomatosis with polyangiitis. *Int J Cardiol.* (2015) 199:170–9. 10.1016/j.ijcard.2015.06.087 26209947

[121] Fijolek J Wiatr E Gawryluk D Nowicka U Martusewicz-Boros M Kober J The significance of cardiac magnetic resonance imaging in detection and monitoring of the treatment efficacy of heart involvement in eosinophilic granulomatosis with polyangiitis patients. *Sarcoidosis Vasc Diffuse Lung Dis.* (2016) 33:51–8. 27055836

[122] Dennert R van Paassen P Schalla S Kuznetsova T Alzand B Staessen J Cardiac involvement in Churg-Strauss syndrome. *Arthritis Rheum.* (2010) 62:627–34. 10.1002/art.27263 20112390

[123] Dunogue B Terrier B Cohen P Marmursztejn J Legmann P Mouthon L Impact of cardiac magnetic resonance imaging on eosinophilic granulomatosis with polyangiitis outcomes: a long-term retrospective study on 42 patients. *Autoimmun Rev.* (2015) 14:774–80. 10.1016/j.autrev.2015.04.013 25960167

[124] Liu S Guo L Zhang Z Li M Zeng X Wang L Cardiac manifestations of eosinophilic granulomatosis with polyangiitis from a single-center cohort in China:clinical features and associated factors. *Ther Adv Chronic Dis.* (2021) 12:1–12. 10.1177/2040622320907051 PMC7841702 33613936

[125] Bischof A Jaeger V Hadden R Luqmani R Probstel A Merkel P Peripheral neuropathy in antineutrophil cytoplasmic antibody-associated vasculitides. Insights from the DCVAS study. *Neurol Neuroimmunol Neuroinflamm.* (2019) 6:615. 10.1212/NXI.000000000000615 PMC6807658 31540965

[126] Wludarczyk A Szczeklik W. Neurological manifestations in ANCA-associated vasculitis – assessment and treatment. *Expert Rev Neurother.* (2016) 16:861–3. 10.1586/14737175.2016.1165095 26967679

[127] Oiwa H Mokuda S Matsubara T Funaki M Takeda I Yamawaki T Neurological complications in eosinophilic granulomatosis with polyangiitis (EGPA): the roles of history and physical examinations in the diagnosis of EGPA. *Int Med.* (2017) 56:3003–8. 10.2169/internalmedicine.8457-16 28924115 PMC5726955

[128] Nishi R Koike H Ohyama K Fukami Y Ikeda S Kawagashira Y Differential clinicopathologic features of EGPA-associated neuropathy with and without ANCA. *Neurology.* (2020) 94:e1726–37. 10.1212/WNL.0000000000009309 32217776

[129] Andre R Cottin V Saraux J Blaison G Bienvenu B Cathebras P Central nervous system involvement in eosinophilic granulomatosis with polyangiitis (Churg-Strauss): report of 26 patients and review of the literature. *Autoimmun Rev.* (2017) 16:963–9. 10.1016/j.autrev.2017.07.007 28709761

[130] Micheletti R Fuxench Z Craven A Watts R Luqmani R Merkel P Cutaneous manifestations of antineutrophil cytoplasmic antibody-associated vasculitis. *Arthritis Rheumatol.* (2020) 72:1741–7. 10.1002/art.41310 32419292

[131] Ishibashi M Kawahara Y Chen K. Spectrum of cutaneous vasculitis in eosinophilic granulomatosis with polyangiitis (Churg-Strauss): a case series. *Am J Dermatopathol.* (2015) 37:214–21. 10.1097/DAD.0000000000000192 25079200

[132] Abdel-Halim M Mahmoud A Ragab G. Cutaneous manifestations of anti-neutrophil cytoplasmic antibody associated vasculitis. *Vessel Plus.* (2022) 6:8. 10.20517/2574-1209.2021.40

[133] Radonjic-Hoesli S Bruggen M Feldmeyer L Simon H Simon D. Eosinophils in skin diseases. *Semin Immunopathol.* (2021) 43:393–409. 10.1007/s00281-021-00868-7 34097126 PMC8241748

[134] Durel C Sinico R Teixeira V Jayne D Belenfant X Marchand-Adam S Renal involvement in eosinophilic granulomatosis with polyangiitis (EGPA): a multicentric retrospective study of 63 biopsy-proven cases. *Rheumatology.* (2020) 0:1–7. 10.1093/rheumatology/keaa416 32856066

[135] Churg A. Recent advances in the diagnosis of Churg-Strauss syndrome. *Mod Pathol.* (2001) 14:1284–93. 10.1038/modpathol.3880475 11743052

[136] Cordier J Cottin V Guillevin L Bel E Bottero P Dalhoff K Eosinophilic granulomatosis with polyangiitis (Churg-Strauss). *Presse Med.* (2013) 42:507–10. 23490637 10.1016/j.lpm.2013.02.308

[137] Masi A Hunder G Lie J Michel B Bloch D Arend W The American College of Rheumatology 1990 criteria for the classification of Churg-Strauss syndrome (allergic granulomatosis and angiitis). *Arthritis Rheum.* (1990) 33:1094–100. 10.1002/art.1780330806 2202307

[138] Grayson P Ponte C Suppiah R Robson J Craven A Judge A 2022 American College of Rheumatology/European Alliance of Associations for Rheumatology Classification Criteria for eosinophilic granulomatosis with polyangiitis. *Arthritis Rheumatol.* (2022) 74:386–92. 10.1002/art.41982 35106968

[139] Guilpain P Auclair J Tamby M Servettaz A Mahr A Weill B Serum eosinophil cationic protein: a marker of disease activity in Churg-Strauss syndrome. *Ann N Y Acad Sci.* (2007) 1107:392–9. 10.1196/annals.1381.041 17804567

[140] Dallos T Heiland G Strehl J Karonitsch T Gross W Moosig F CCL17/thymus and activation – related chemokine in Churg-Strauss syndrome. *Arthritis Rheum.* (2010) 62:3496–503. 10.1002/art.27678 20669282

[141] Grayson P Monach P Pagnoux C Cuthbertson D Carette S Hoffman G Values of commonly measured laboratory tests as biomarkers of disease activity and predictors of relapse in eosinophilic granulomatosis with polyangiitis. *Rheumatology.* (2015) 54:1351–9. 10.1093/rheumatology/keu427 25406357 PMC4502335

[142] Rhee R Holweg C Wong K Cuthbertson D Carette S Khalidi N Serum periostin as a biomarker in eosinophilic granulomatosis with polyangiitis. *PLoS One.* (2018) 13:e0205768. 10.1371/journal.pone.0205768 30308057 PMC6181402

[143] Pagnoux C Nair P Xi Y Khalidi N Carette S Cuthbertson D Serum cytokine and chemokine levels in patients with eosinophilic granulomatosis with polyangiitis, hypereosinophilic syndrome, or eosinophilic asthma. *Clin Exp Rheumatol.* (2019) 37(Suppl. 117):40–4. PMC9878582 30652675

[144] Laskari K Hellmich B Adamus G Csernok E. Autoantibody profile in eosinophilic granulomatosis and polyangiitis: predominance of anti-alpha-enolase antibodies. *Clin Exp Rheumatol.* (2021) 39(Suppl.129):S83–7. 10.55563/clinexprheumatol/08k9af 33200729

[145] Rodriguez-Pla A Warner R Cuthbertson D Carette S Khalidi N Koening C Evaluation of potential serum biomarkers of disease activity in diverse forms of vasculitis. *J Rheumatol.* (2020) 47:1001–10. 10.3899/jrheum.190093 31474593 PMC7050393

[146] Padoan R Gatto M Ghirardello A Tonello M Franco C Felicetti M IgG anti-pentraxin 3 antibodies are a novel biomarker of ANCA-associated vasculitis and better identify patients with eosinophilic granulomatosis with polyangiitis. *J Autoimmun.* (2021) 124:102725. 10.1016/j.jaut.2021.102725 34534841

[147] Szczeklik W Sanak M Mastalerz L Sokolowska B Gielicz A Soja J 12-hydroxy-eicosatetraenoic acid (12-HETE): a biomarker of Churg-Strauss syndrome. *Clin Exp Allergy.* (2012) 42:513–22. 10.1111/j.1365-2222.2011.03943.x 22417211

[148] Ma J Dong C Wei S Qiu M Wu P Ou C Serum cytokine profiling identifies Axl as a new biomarker candidate for active eosinophilic granulomatosis with polyangiitis. *Front Mol Biosci.* (2021) 8:653461. 10.3389/fmolb.2021.653461 33987203 PMC8112820

[149] Latorre M Bacci E Seccia V Dente F Brighindi S Di Franco A New biomarkers for early diagnosis of eosinophilic granulomatosis with polyangiitis (EGPA). *Euro Respir J.* (2017) 50:A3560. 10.1183/1393003 31692595

[150] Xiao J Lu S Wang X Liang M Dong C Zhang X Serum proteomic analysis identifies SAA1, FGA, SAP, and CETP as new biomarkers for eosinophilic granulomatosis with polyangiitis. *Front Immunol.* (2022) 13:866035. 10.3389/fimmu.2022.866035 35757752 PMC9226334

[151] Zhao B Zheng H Yang T Zheng R. Eosinophilic granulomatosis with polyangiitis in allergic asthma: efforts to make early diagnosis possible. *Allergy Asthma Proc.* (2023) 44:59–63. 10.2500/aap.2023.44.220072 36719697

[152] Moiseev S Bossuyt X Arimura Y Blockmans D Csernok E Damoiseaux J International Consensus on antineutrophil cytoplasm antibodies testing in eosinophilic granulomatosis with polyangiitis. *Am J Respir Crit Care Med.* (2020) 202:1360–72. 10.1164/rccm.202005-1628SO 32584187

[153] Groh M Pagnoux C Baldini C Bel E Bottero P Cottin V Eosinophilic granulomatosis with polyangiitis (Churg-Strauss) (EGPA). Consensus Task Force recommendations for evaluation and management. *Eur J Int Med.* (2015) 26:545–53. 10.1016/j.ejim.2015.04.022 25971154

[154] Guillevin L Lhote F Gayraud M Cohen P Jarrousse B Lortholary O Prognostic factors in polyarteritis nodosa and Churg-Strauss syndrome. A prospective study in 342 patients. *Medicine.* (1996) 75:17–28. 10.1097/00005792-199601000-00003 8569467

[155] Bond M Fagni F Moretti M Bello F Egan A Vaglio A At the heart of eosinophilic granulomatosis with polyangiitis: into cardiac and vascular involvement. *Cur Rheumatol Rep.* (2022) 24:337–51. 10.1007/s11926-022-01087-1 36194339

[156] Cereda A Pedrotti P De Capitani L Giannattasio C Roghi A. Comprehensive evaluation of cardiac involvement in eosinophilic granulomatosis with polyangiitis (EGPA) with cardiac magnetic resonance. *Eur J Int Med.* (2017) 39:51–6. 10.1016/j.ejim.2016.09.014 27727077

[157] Mavrogeni S Dimitroulas T Kitas G. Cardiovascular magnetic resonance in the diagnosis and management of cardiac and vascular involvement in the systemic vasculitides. *Curr Opin Rheumatol.* (2019) 31:16–24. 30407225 10.1097/BOR.0000000000000560

[158] Yune S Choi D Lee B Lee J Jeon E Kim S Detecting cardiac involvement with magnetic resonance in patients with active eosinophilic granulomatosis with polyangiitis. *Int J Cardiovasc Imaging.* (2016) 32(Suppl 1):S155–62. 10.1007/s10554-016-0843-y 26831057

[159] Fijolek J Wiatr E Blasinska K Piotrowska-Kownacka D Roszkowski-Sliz K. Cardiac involvement and absence of asthma-what is phenotype specificity of EGPA: a case report. *Clin Med Res.* (2022) 20:170–6. 10.3121/cmr.2022.1683

[160] Marmursztejn J Vignaux O Cohen P Guilpain P Pagnoux C Gouya H Impact of cardiac magnetic resonance imaging for assessment of Churg-Strauss syndrome: a cross-sectional study in 20 patients. *Clin Exp Rheumatol.* (2009) 27(Suppl. 52):S70–6. 19646350

[161] Miszalski-Jamka T Szczeklik W Sokolowska B Karwat K Belzak K Mazur W Standard and feature tracking magnetic resonance evidence of myocardial involvement in Churg-Strauss syndrome and granulomatosis with polyangiitis (Wegener’s) in patients with normal electrocardiograms and transthoracic echocardiography. *Int J Cardiovasc Imaging.* (2013) 29:843–53. 10.1007/s10554-012-0158-6 23212274 PMC3644401

[162] Nasser M Thivolet-Bejui F Seve P Cottin V. Lung-limited or lung-dominant variant of eosinophilic granulomatosis with polyangiitis. *J Allergy Clin Immunol Pract.* (2020) 8:2092–5. 10.1016/j.jaip.2020.01.058 32061719

[163] Taylor M Keane C O’Connor P Mulvihill E Holland C. The expanded spectrum of toxocaral disease. *Lancet.* (1988) 1:692–5. 10.1016/s0140-6736(88)91486-9 2895221

[164] Siddiqui A Berk S. Diagnosis of Strongyloides stercoralis infection. *Clin Infect Dis.* (2001) 33:1040–7. 10.1086/322707 11528578

[165] Kahn J Groh M Lefevre G. (A critical appraisal of) classification of hypereosinophilic disorders. *Front Immunol.* (2016) 4:216. 10.3389/fmed.2017.00216 29259972 PMC5723313

[166] Asano K Hebisawa A Ishiguro T Takayanagi N Nakamura Y Suzuki Y New clinical diagnostic criteria for allergic bronchopulmonary aspergillosis/mycosis and its validation. *J Allergy Clin Immunol.* (2021) 147:1261–8. 10.1016/j.jaci.2020.08.029 32920094

[167] Crowe M Robinson D Sagar M Chen L Ghamande S. Chronic eosinophilic pneumonia: clinical perspectives. *Ther Clin Risk Manag.* (2019) 15:397–403. 10.2147/TCRM.S157882 30936702 PMC6420789

[168] Khoury P Zagallo P Talar-Williams C Santos C Dinerman E Holland N Serum biomarkers are similar in Churg-Strauss syndrome and hypereosinophilic syndrome. *Allergy.* (2012) 67:1149–56. 10.1111/j.1398-9995.2012.02873.x 22775568 PMC3418460

[169] Shomali W Gotlib J. World Health Organization-defined eosinophilic disorders: 2022 update on diagnosis, risk stratification, and management. *Am J Hematol.* (2022) 97:129–48. 10.1002/ajh.26352 34533850

[170] Groh M Kahn J Puechal X Guillevin L. Comment on: first report of FIP1L1-PDGFRalpha-positive eosinophilic granulomatosis with polyangiitis. *Rheumatology.* (2016) 55:384–5. 10.1093/rheumatology/kev425 26647460

[171] Leru P. Eosinophilic disorders: evaluation of current classification and diagnostic criteria, proposal of a practical diagnostic algorithm. *Clin Transl Allergy.* (2019) 9:36. 10.1186/s13601-019-0277-4 31367340 PMC6657042

[172] Curtis C Ogbogu P. Hypereosinophilic syndrome. *Clin Rev Allergy Immunol.* (2016) 50:240–51. 10.1007/s12016-015-8506-7 26475367

[173] Wei X Li X Wei Z Zhang H Deng J Xing S Clinical analysis of hypereosinophilic syndrome first presenting with asthma-like symptoms. *Ann Med.* (2022) 54:11–21. 10.1080/07853890.2021.2014555 34935570 PMC8725856

[174] Lefevre G Leurs A Gibier J Copin M Staumont-Salle D Dezoteux F “Idiopathic eosinophilic vasculitis”: Another side of hypereosinophilic syndrome? A comprehensive analysis of 117 cases in asthma-free patients. *J Allergy Clin Immunol Pract.* (2020) 8:1329–40.e3. 10.1016/j.jaip.2019.12.011 31863912

[175] Leurs A Chenivesse C Lopez B Gibier J Clement G Groh M C-reactive protein as a diagnostic tool in differential diagnosis of hypereosinophilic syndrome and ANCA-negative eosinophilic granulomatosis with polyangiitis. *J Allergy Clin Immunol Pract.* (2019) 7:1347–51.e3. 10.1016/j.jaip.2018.10.002 30317003

[176] Ahn S Yoo J Park Y Park J Lee J Lee S. A new index for distinguishing hypereosinophilic syndrome and antineutrophil cytoplasmic antibody-negative eosinophilic granulomatosis with polyangiitis. *Asian Pac J Allergy Immunol.* (2020). 10.12932/AP-080420-0805 33068368

[177] Takahashi H Komai T Setoguchi K Shoda H. A diagnostic score for eosinophilic granulomatosis with polyangiitis among eosinophilic disorders. *Allergol Int.* (2023) 72:316–23. 10.1016/j.alit.2022.08.008 36184347

[178] Puechal X. Granulomatosis with polyangiitis (Wegener’s). *Joint Bone Spine.* (2020) 87:572–8. 10.1016/j.jbspin.2020.06.005 32562697

[179] Greco A De Virgilio A Rizzo M Gallo A Magliulo G Fusconi M Microscopic polyangiitis: advances in diagnostic and therapeutic approaches. *Autoimmun Rev.* (2015) 14:837–44. 10.1016/j.autrev.2015.05.005 25992801

[180] Nguyen Y Pagnoux C Karras A Quemeneur T Maurier F Hamidou M Microscopic polyangiitis: clinical characteristics amd long-term outcomes of 378 patients from the French Vasculitis Study Group Registry. *J Autoimmun.* (2020) 112:102467. 10.1016/j.aut.2020.102467 32340774

[181] Hernandez-Rodriguez J Alba M Prieto-Gonales S Cid M. Diagnosis and classification of polyarteritis nodosa. *J Autoimmun.* (2014) 48-49:84–9. 10.1016/j.jaut.2014.01.029 24485157

[182] Morales A Cignarella A Jabeen I Barkin J Mirsaeidi M. An update on IgG4-related lung disease. *Eur J Int Med.* (2019) 66:18–24. 10.16/j.ejim.2019.06.010 31227290

[183] Simo-Perdigo M Martinez-Valle F. IgG4-related disease. *Rev Esp Med Nucl Imagen Mol. (Engl Ed).* (2021) 40:107–14. 10.1016/j.remn.2020.12.001 33547020

[184] Chang H Chou P Lai C Tsai H. Antineutrophil cytoplasmic antibodies and organ-specific manifestations on eosinophilic granulomatosis with polyangiitis: a systematic review and meta-analysis. *J Allergy Clin Immunol Pract.* (2021) 9:445–52. 10.1016/j.jaip.2020.07.038 32771687

[185] Liu S Han L Liu Y Yang J Zhang Y Li M Clinical significance of MPO-ANCA in eosinophilic granulomatosis with polyangiitis: experience from a longitudinal Chinese cohort. *Front Immunol.* (2022) 13:885198. 10.3389/fimmu.2022.885198 35833130 PMC9271578

[186] Papo M Sinico R Teixeira V Venhoff N Urban M Iudici M Significance of PR3-ANCA positivity in eosinophilic granulomatosis with polyangiitis (Churg-Strauss). *Rheumatology.* (2020) 60:4355–60. 10.1093/rheumatology/keaa805 33347592

[187] Terrier B Darbon R Durel C Hachulla E Karras A Maillard H French recommendations for the management of systemic necrotizing vasculitides (polyarteritis nodosa and ANCA-associated vasculitides). *Orphanet J Rare Dis.* (2020) 15:351. 10.1186/s13023-020-01621-3 33372616 PMC7771069

[188] Goglin S Chung S. New developments in treatments for systemic vasculitis. *Cur Opin Pharmacol.* (2022) 66:102270. 10.116/j.coph.2022.102270 35921775

[189] Asano K Suzuki Y Tanaka J Kobayashi K Kamide Y. Treatments of refractory eosinophilic lung diseases with biologics. *Allergol Int*. (2023) 72:31–40. 10.1016/j.alit.2022.10.004 36333218

[190] Raffray L Guillevin L. Updated for the treatment of EGPA. *Presse Med.* (2020) 49:104036. 10.1016/j.lpm.2020.104036 32652104

[191] Ribi C Cohen P Pagnoux C Mahr A Arene A Lauque J Treatment of Churg-Strauss syndrome without poor-prognosis factors: a multicenter, prospective, randomized, open – label study of seventy-two patients. *Arthritis Rheum.* (2008) 58:586–94. 10.1002/art.23198 18240234

[192] Mukhtyar C Guillevin L Cid M Dasgupta B de Groot K Gross W EULAR recommendations for the management of primary small and medium vessel vasculitis. *Ann Rheum Dis.* (2009) 68:310–7. 10.1136/ard.2008.088096 18413444

[193] Puechal X Pagnoux C Baron G Quemeneur T Neel A Agard C Adding azathioprine to remission-induction glucocorticoids for eosinophilic granulomatosis with polyangiitis (Churg-Strauss), microscopic polyangiitis, or polyarteritis nodosa without poor prognosis factors: a randomized, controlled trial. *Arthritis Rheumathol.* (2017) 69:2175–86. 10.1002/art.40205 28678392

[194] Chung S Langford C Maz M Abril A Gorelik M Guyatt G 2021 American college of rheumatology/vasculitis foundation guidelines for the management of antineutrophil cytoplasmic antibody-associated vasculitis. *Arthritis Rheumatol.* (2021) 0:1–18. 10.1002/art.41773 34235894 PMC12327957

[195] Kim S Marigowda G Oren E Israel E Wechsler M. Mepolizumab as a steroid –sparing treatment option in patients with Churg-Strauss syndrome. *J Allergy Clin Immunol.* (2010) 125:1336–43. 10.1016/j.jaci.2010.03.028 20513524

[196] Moosig F Gross W Hermann K Bremer J Hellmich B. Targeting interleukin-5 in refractory and relapsing Churg-Strauss syndrome. *Ann Intern Med.* (2011) 155:341–3. 10.7326/0003-4819-155-5-201109060-00026 21893636

[197] Steinfeld J Bradford E Brown J Mallett S Yancey S Akuthota P Evaluation of clinical benefit from treatment with mepolizumab for patients with eosinophilic granulomatosis with polyangiitis. *J Allergy Clin Immunol.* (2019) 143:2170–7. 10.1016/j.jaci.2018.11.041 30578883 PMC7254609

[198] Uzzo M Regola F Trezzi B Toniati P Franceschini F Sinico R. Novel targets for drug use in eosinophilic granulomatosis with polyangiitis. *Front Med.* (2021) 8:754434. 10.3389/fmed.2021.754434 34796188 PMC8593004

[199] Khoury P Akuthota P Baylis L Chang S Bentley J Wechsler M. Impact of baseline treatment, duration of disease, and refractory status on outcomes in mepolizumab-treated patients with EGPA. *J Allergy Clin Immunol.* (2022) 149(Suppl.):AB52.

[200] Terrier B Charles P Aumaitre O Belot A Bonnotte B Crabol Y ANCA-associated vasculitides: recommendations of the French Vasculitis Study Group on the use of immunosuppressants and biotherapies for remission induction and maintenance. *Presse Med.* (2020) 49:104031. 10.1016/j.Jpm.2020.104031 32645418

[201] Detoraki A Tremante E Poto R Morelli E Quaremba G Granata F Real-life evidence of low-dose mepolizumab efficacy in EGPA: a case series. *Respir Res.* (2021) 22:185. 10.1186/s12931-021-01775-z 34162391 PMC8220666

[202] Vultaggio A Nencini F Bormioli S Vivarelli E Dies L Rossi O Low-dose mepolizumab effectiveness in patients suffering from eosinophilic granulomatosis with polyangiitis. *Allergy Asthma Immunol Res.* (2020) 12:885–93. 10.4168/aair.2020.12.5.885 32638567 PMC7346991

[203] Özdel Öztürk B Yavuz Z Aydin Ö Mungan D Sin B Demirel Y Effectiveness of low-dose mepolizumab in the treatment of eosinophilic granulomatosis with polyangiitis (EGPA): a real-life experience. *Int Arch Allergy Immunol.* (2022) 183:1280–90. 10.1159/000526410 36126640

[204] Bettiol A Urban M Dagna L Cottin V Franceschini F Del Giacco S Mepolizumab for eosinophilic granulomatosis with polyangiitis: a European multicenter observational study. *Arthritis Rheumatol.* (2022) 74:295–306. 10.1002/art.41943 34347947 PMC9305132

[205] Ueno M Miyagawa I Nakano K Iwata S Hanami K Fukuyo S Effectiveness and safety of mepolizumab in combination with corticosteroids in patients with eosinophilic granulomatosis with polyangiitis. *Arthritis Res Ther.* (2021) 23:86. 10.1186/s13075-021-02462-6 33726827 PMC7962235

[206] Manka L Guntur V Denson J Dunn R Dollin Y Strand M Efficacy and safety of reslizumab in the treatment of eosinophilic granulomatosis with polyangiitis. *Ann Allergy Asthma Immunol.* (2021) 126:696–701. 10.1016/j.anai.2021.01.035 33548468

[207] Guntur V Manka L Denson J Dunn R Dollin Y Gill M Benralizumab as a steroid-sparing treatment option in eosinophilic granulomatosis with polyangiitis. *J Allergy Clin Immunol Pract.* (2021) 9:1186–93.e1. 10.1016/j.jaip.2020.09.054 33065367

[208] Steinke J Borish L. Th2 cytokines and asthma. Interleukin-4: its role in the pathogenesis of asthma, and targeting it for asthma treatment with interleukin-4 receptor antagonists. *Respir Res.* (2001) 2:66–70. 10.1186/rr40 11686867 PMC59570

[209] Castro M Corren J Pavord I Maspero J Wenzell S Rabe K Dupilumab efficacy and safety in moderate-to-severe uncontrolled asthma. *N Engl J Med.* (2018) 378:2486–96. 10.1056/NEJMoa1804092 29782217

[210] Wechsler M Ford L Maspero J Pavord I Papi A Bourdin A Long-term safety and efficacy of dupilumab in patients with moderate-to-severe asthma (TRAVERSE): an open-label extension study. *Lancet Respir Med.* (2022) 10:11–25. 10.1016/S2213-2600(21)00322-2 34597534

[211] Wechsler M Ruddy M Pavord I Israel E Rabe K Ford L Efficacy and safety of itepekimab in patients with moderate-to-severe asthma. *N Engl J Med.* (2021) 385:1656–68. 10.1056/NEJMoa2024257 34706171

[212] Menzies-Gow A Colice G Griffiths J Almqvist G Ponnarambil S Kaur P NAVIGATOR: a phase 3 multicentre, randomized, double-blind, placebo-controlled, paralel-group trial to evaluate the efficacy and safety of tezepelumab in adults and adolescents with severe, uncontrolled asthma. *Respir Res.* (2020) 21:266. 10.1186/s12931-020-01526-6 33050934 PMC7550847

[213] Siddiqui S Bozik M Archibald D Dworetzky S Mather J Killingsworth R Late breaking abstract – phase 2 trial evaluating the effects of dexpramipexole on blood eosinophils, lung function, and airway biomarkers in eosinophilic asthma. *Eur Respir J.* (2021) 58(Suppl. 65):RCT2900. 10.1183/13993003

[214] Panch S Bozik M Brown T Makiya M Prussin C Archibald D Dexpramipexole as an oral steroid-sparing agent in hypereosinophilic syndromes. *Blood.* (2018) 132:501–9. 10.1182/blood-2018-02-835330 29739754 PMC6073324

[215] Jones R Tervaert J Hauser T Luqmani R Morgan M Peh C Rituximab versus cyclophosphamide in ANCA-associated renal vasculitis. *N Engl J Med.* (2010) 363:211–20. 10.1056/NEJMoa0909169 20647198

[216] Terrier B Pagnoux C Perrodeau E Karras A Khouatra C Aumaitre O Long-term efficacy of remission – maintenance regimens for ANCA-associated vasculitides. *Ann Rheum Dis.* (2018) 77:1150–6. 10.1136/annrheumdis-2017-212768 29724729

[217] Mohammad A Hot A Arndt F Moosig F Guerry M Amudala N Rituximab for the treatment of eosinophilic granulomatosis with polyangiitis (Churg-Strauss). *Ann Rheum Dis.* (2016) 75:396–401. 10.1136/annrheumdis-2014-206095 25467294

[218] Teixeira V Mohammad A Jones R Smith R Jayne D. Efficacy and safety of rituximab in the treatment of eosinophilic granulomatosis with polyangiitis. *RMD Open.* (2019) 5:e000905. 10.1136/rmdopen-2019-000905 31245051 PMC6560673

[219] Canzian A Venhoff N Urban M Sartorelli S Ruppert A Groh M Use of biologics to treat relapsing and/or refractory eosinophilic granulomatosis with polyangiitis: data from a European Collaborative Study. *Arthritis Rheumatol.* (2021) 73:498–503. 10.1002/art.41534 33001543

[220] Terrier B Pugnet G de Moreuil C Bonnotte B Benhamou Y Diot E Rituximab versus conventional therapeutic strategy for remission induction in eosinophilic granulomatosis with polyangiitis: a double-blind, randomized, controlled trial. *Arthritis Rheumatol.* (2021) 73 (Suppl. 9). Available online at: https://acrabstracts.org/abstract/rituximab-versus-conventional-therapeutic-strategy-for-remission-induction-in-eosinophilic-granulomatosis-with-polyangiitis-a-double-blind-randomized-controlled-trial/

[221] Cheungpasitporn W Kopecky S Specks U Bharucha K Fervenza C. Non-ischemic cardiomyopathy after rituximab treatment for membranous nephropathy. *J Renal Inj Prev.* (2017) 6:18–25. 10.15171/jrip.2017.04 28487867 PMC5414514

[222] Pepper R Fabre M Pavesio C Gaskin G Jones R Jayne D Rituximab is effective in the treatment of refractory Churg-Strauss syndrome and is associated with diminished T-cell interleukin-5 production. *Rheumatology.* (2008) 47:1104–5. 10.1093/rheumatology/ken175 18492710

[223] Moura M Berti A Keogh K Volcheck G Specks U Baqir M. Asthma control in eosinophilic granulomatosis with polyangiitis treated with rituximab. *Clin Rheumatol.* (2020) 39:1581–90. 10.1007/s10067-019-04891-w 31897956

[224] Higashitani K Yoshimi R Sato Y Watanabe T Ihata A. Rituximab and mepolizumab combination therapy for glucocorticoid-resistant myocarditis related to eosinophilic granulomatosis with polyangiitis. *Mod Rheumatol Case Rep.* (2022) 6:87–92. 10.1093/mrcr/rxab022 34473835

[225] Afiari A Gabriel A Gaiki M. Concurrent use of mepolizumab and rituximab for eosinophilic granulomatosis with polyangiitis and multisystem involvement. *Cureus.* (2020) 12:e9242. 10.7759/cureus.9242 32821588 PMC7430662

[226] Bettiol A Urban M Bello F Fiori D Mattioli I Lopalco G Sequential rituximab and mepolizumab in eosinophilic granulomatosis with polyangiitis (EGPA): a European multicenter observational study. *Ann Rheum Dis.* (2022) 81:1769–72. 10.1136/annrheumdis-2022-222776 35850947

[227] Basta F Mazzuca C Nucera E Schiavino D Afeltra A Incalzi R. Omalizumab in eosinophilic granulomatosis with polyangiitis: friend or foe? A systematic literature review. *Clin Exp Rheumatol.* (2020) 38(Suppl 124):S214–20. 32083537

[228] Celebi Sozener Z Gorgulu B Mungan D Sin B Misirligil Z Aydin O Omalizumab in the treatment of eosinophilic granulomatosis with polyangiitis (EGPA): single-center experience in 18 cases. *World Allergy Organ J.* (2018) 11:39. 10.1186/s40413-018-0217-0 30524647 PMC6276141

[229] Iglesias E Camacho Lovillo M Delgado Pecellin I Lirola Cruz M Falcon Neyra M Salazar Quero J Successful management of Churg-Strauss syndrome using omalizumab as adjuvant immunomodulatory therapy: first documented pediatric case. *Pediatr Pulmonol.* (2014) 49:E78–81. 10.1002/ppul.22884 24136903

[230] Nazir S Tachamo N Fareedy S Khan M Lohani S. Omalizumab – associated eosinophilic granulomatosis with polyangiitis (Churg-Strauss syndrome). *Ann Allergy Asthma Immunol.* (2017) 118:372–4. 10.1016/j.anai.2016.12.003 28094120

[231] Metzler C Schnabel A Gross W Hellmich B. A phase II study of interferon-alpha for the treatment of refractory Churg-Strauss syndrome. *Clin Exp Rheumatol.* (2008) 26(Suppl. 49):S35–40. 18799051

[232] Koike H Akiyama K Saito T Sobue G Research Group for IVIg for Egpa/Css in Japan. Intravenous immunoglobulin for chronic residua peripheral neuropathy in eosinophilic granulomatosis with polyangiitis (Churg-Strauss syndrome): a multicenter, double-blind trial. *J Neurol.* (2015) 262:757–9. 10.1007/s00415-014-7618-y 25577176 PMC4363522

[233] Pecoraro A Crescenzi L Carucci L Genovese A Spadaro G. Heart failure not responsive to standard immunosuppressive therapy is successfully treated with high dose intravenous immunoglobulin therapy in a patient with eosinophilic granulomatosis with polyangiitis (EGPA). *Int Immunopharmacol.* (2017) 45:13–5. 10.1016/j.intimp.2017.01.025 28152445

[234] Walsh M Merkel P Peh C Szpirt W Puechal X Fujimoto S Plasma exchange and glucocorticoids in severe ANCA-associated vasculitis. *N Engl J Med.* (2020) 382:622–31. 10.1056/NEJMoa1803537 32053298 PMC7325726

[235] Guillevin L Lhote E Cohen P Jarrousse B Lortholary O Genereau T Corticosteroids plus pulse cyclophosphamide and plasma exchanges versus corticosteroids plus pulse cyclophosphamide alone in the treatment of polyarteritis nodosa and Churg-Strauss syndrome patients with factors predicting poor prognosis. A prospective, randomized trial in sixty-two patients. *Arthritis Rheum.* (1995) 38:1638–45. 10.1002/art.1780381116 7488285

[236] Global Initiative for Asthma [GINA]. *2022 GINA Report: Global Strategy for Asthma Management and Prevention.* (n.d.). Available online at: https://ginasthma.org/wp-content/uploads/2022/07/GINA-Main-Report-2022-FINAL-22-07-01-WMS.pdf (accessed July 7, 2022).

[237] Baldwin C Wolter N Pagnoux C. Ear, nose, and throat involvement in eosinophilic granulomatosis with polyangiitis. *Adv Cell Mol Otolaryngol.* (2015) 3:2781. 10.3402/acmo.v3.27181

[238] Padoan R Campaniello D Felicetti M Cazzador D Schiavon F. Ear, nose, and throat in ANCA-associated vasculitis: a comprehensive review. *Vessel Plus.* (2021) 5:41. 10.20517/2574-1209.2021.41

[239] Han J Bachert C Fokkens W Desrosiers M Wagenmann M Lee S Mepolizumab for chronic rhinosinusitis with nasal polyps (SYNAPSE): a randomized, double-blind, placebo-controlled, phase 3 trial. *Lancet Respir Med.* (2021) 9:1141–53. 10.1016/S2213-2600(21)00097-7 33872587

[240] Pendolino A Unadkat S Zhang H Pendolino M Bianchi G Randhawa P The role of surgery in antineutrophil cytoplasmic antibody-associated vasculitides affecting the nose and sinuses: a systematic review. *SAGE Open Med.* (2020) 8:1–12. 10.1177/2050312120936731 32676189 PMC7340348

[241] Doubelt I Cuthbertson D Carette S Chung SA Forbess LJ Khalidi NA Clinical manifestations and long-term outcomes of eosinophilic granulomatosis with polyangiitis in North America. *ACR Open Rheumatol.* (2021) 3:404–12. 10.1002/acr2.11263 34032390 PMC8207688

[242] Kim D Song J Park Y Lee S. Five factor score of more than 1 is associated with relapse during the first 2 year – follow up in patients with eosinophilic granulomatosis with polyangiitis. *Int J Rheum Dis.* (2017) 20:1261–8. 10.1111/1756-185X.13056 28261989

[243] Alberici F Bonatti F Adorni A Daminelli G Sinico R Gregorini G FCGR3B polymorphism predicts relapse risk in eosinophilic granulomatosis with polyangiitis. *Rheumatology.* (2020) 59:3563–6. 10.1093/rheumatology/keaa134 32375167

[244] Keogh K Specks U. Churg-Strauss syndrome: clinical presentation, antineutrophil cytoplasmic antibodies, and leukotriene receptor antagonists. *Am J Med.* (2003) 115:284–90. 10.1016/s0002-9343(03)00359-0 12967693

[245] King C Harper L Little M. The complications of vasculitis and its treatment. *Best Pract Res Clin Rheumatol.* (2018) 32:125–36. 10.1016/j.berh.2018.07.009 30526892

[246] Puechal X Pagnoux C Baron G Lifermann F Geffray L Quemeneur T Non-severe eosinophilic granulomatosis with polyangiitis: long-term outcomes after remission-induction trial. *Rheumatology.* (2019) 58:2107–16. 10.1093/rheumatology/kez139 31056661

[247] Bell C Blauer-Peterson C Mao J. Burden of illnes and costs associated with eosinophilic granulomatosis with polyangiitis: evidence from a managed care database in the United States. *J Manag Care Spec Pharm.* (2021) 27:1249–59. 10.18553/jmcp.2021.21002 34165321 PMC10394225

